# Inter-Subject Correlation in fMRI: Method Validation against Stimulus-Model Based Analysis

**DOI:** 10.1371/journal.pone.0041196

**Published:** 2012-08-08

**Authors:** Juha Pajula, Jukka-Pekka Kauppi, Jussi Tohka

**Affiliations:** 1 Department of Signal Processing, Tampere University of Technology, Tampere, Finland; 2 Department of Computer Science, University of Helsinki, Helsinki, Finland; 3 Department of Signal Processing, Tampere University of Technology, Tampere, Finland; The University of Melbourne, Australia

## Abstract

Within functional magnetic resonance imaging (fMRI), the use of the traditional general linear model (GLM) based analysis methods is often restricted to strictly controlled research setups requiring a parametric activation model. Instead, Inter-Subject Correlation (ISC) method is based on voxel-wise correlation between the time series of the subjects, which makes it completely non-parametric and thus suitable for naturalistic stimulus paradigms such as movie watching. In this study, we compared an ISC based analysis results with those of a GLM based in five distinct controlled research setups. We used International Consortium for Brain Mapping functional reference battery (FRB) fMRI data available from the Laboratory of Neuro Imaging image data archive. The selected data included measurements from 37 right-handed subjects, who all had performed the same five tasks from FRB. The GLM was expected to locate activations accurately in FRB data and thus provide good grounds for investigating relationship between ISC and stimulus induced fMRI activation. The statistical maps of ISC and GLM were compared with two measures. The first measure was the Pearson's correlation between the non-thresholded ISC test-statistics and absolute values of the GLM Z-statistics. The average correlation value over five tasks was 0.74. The second was the Dice index between the activation regions of the methods. The average Dice value over the tasks and three threshold levels was 0.73. The results of this study indicated how the data driven ISC analysis found the same foci as the model-based GLM analysis. The agreement of the results is highly interesting, because ISC is applicable in situations where GLM is not suitable, for example, when analyzing data from a naturalistic stimuli experiment.

## Introduction

Inter-subject correlation (ISC) analysis method provides an opportunity for the functional magnetic resonance imaging (fMRI) analysis under naturalistic research paradigms. In these paradigms, the stimuli are designed to be closer to normal everyday life than in conventional research paradigms. The used stimuli can be, for example, a movie or a 3D video game [1].

One of the major benefits of the ISC analysis is that it can be used to locate activations without *a priori* knowledge of the temporal composition of processes contributing to the neuronal activation. In the ISC analysis, the hemodynamic activity of a subject is used to quantify the hemodynamic activity of another subject by calculating the correlation coefficient between the corresponding fMRI time series of the subjects. Inferences about the locations of activations are solely based on the similarities in hemodynamic responses across the subjects. Instead, a massively univariate stimulus-model-based analysis in fMRI predominantly relies on the theory of general linear models that provide a framework of analyzing subjects fMRI responses with respect to the model of the known and fixed stimulus type, typically appearing as the columns of the design (or predictor) matrix in the GLM. This often restricts the application of these GLM-based analyses to strictly controlled research setups as the parametric model for the BOLD signal changes related to the activation have to be defined *a priori*. The major difference between ISC and GLM based analyses is that the former is completely non-parametric in the sense it does not require any parametric form for the stimulus time-course while the latter requires a model for the stimulus time course. We note that there is a direct connection between the statistical analysis of a slope parameter in a simple regression, i.e., a simplified version of a single subject GLM-based analysis and a correlation coefficient. In what follows, we will use the terms ISC and GLM analysis rather loosely, referring to the major difference explained above rather than to the technical details of computations and statistics involved.

Hasson et al. [2] introduced the concept of ISC in fMRI and demonstrated that a simple movie stimulus produced significant correlations between the voxel-wise fMRI time series of the subjects, especially in visual and auditory cortices. Since then ISC analysis has been applied to investigate speech comprehension [3], auditory abnormalities [4], memory encoding [5] and brain functions during movie watching [2], [6]–[8]. In a particular relation to this work, Kauppi et al. [9] developed a new ISC based method by adding an option to compute the frequency specific ISC and designed novel non-parametric resampling tests to make inferences about ISCs. Resampling tests were designed, since the data was not guaranteed to be uncorrelated as Heijnar et al. [4] had earlier noted. Significant ISCs were found in visual and auditory areas in line with earlier neurocinematics studies and additionally in pre-frontal cortical areas when studying low frequency bands.

One of the main questions concerning the ISC analysis is how to interpret correlations between subjects. Because the ISC measures the similarity of subjects' Blood Oxygenation Level Dependent (BOLD) fMRI responses during the same stimulus, a high ISC does not directly imply a high degree of task (or stimulus) related activation [4]. However, it has been shown by comparing intracranial single-unit and local field potential recordings of epilepsy patients and fMRI of healthy subjects experiencing the same movie stimulus that the correlated firing rate in a local population of neurons correlates with the BOLD response ([10], [11]). Further, Hanson et al. [12] argued that if there are correlations between individual subjects, who all experience the same stimuli, most of the correlated activations should be caused by the stimuli and so it might be possible to find the activity patterns of the brain even in complex situations.

A parametric GLM-based analysis is a standard method for detecting task-related activations in fMRI. Therefore, a potential way to investigate whether an ISC analysis method can locate activated brain regions due to stimulus presentation is to compare the results of the ISC analysis with those of the GLM analysis for the same fMRI data.

In this case, the data must be acquired under strictly controlled experimental setting so that the GLM analysis can be performed reliably. Previously, Heijnar et al. [4] studied ISCs of 20 subjects with the fMRI data acquired during the auditory oddball task and compared the results with those of the GLM. Multi-subject ISC maps were thresholded empirically, as it was noted that statistical thresholds cannot be obtained using standard statistical approaches due to dependencies between the correlations. The comparison was limited to the visual analysis of the activation maps. The conclusion was that the ISC analysis could find the same activation foci as GLM but ISC also found foci which were not visible in the model-based results.

Also in this work, we compare the ISC analysis results with those of the model-based GLM method to investigate the accuracy of the non model-based ISC analysis method detecting activated brain regions. We considerably extend the study of Hejnar et al. by incorporating more tasks and subjects to the comparative analysis. Moreover, we evaluate the similarity of the analysis results quantitatively and use a resampling-based method to obtain statistical thresholds for the ISC brain maps. It is important to use automatic thresholding scheme instead of a manual threshold selection to avoid a possible user-dependent bias in the comparison.

**Figure 1:**
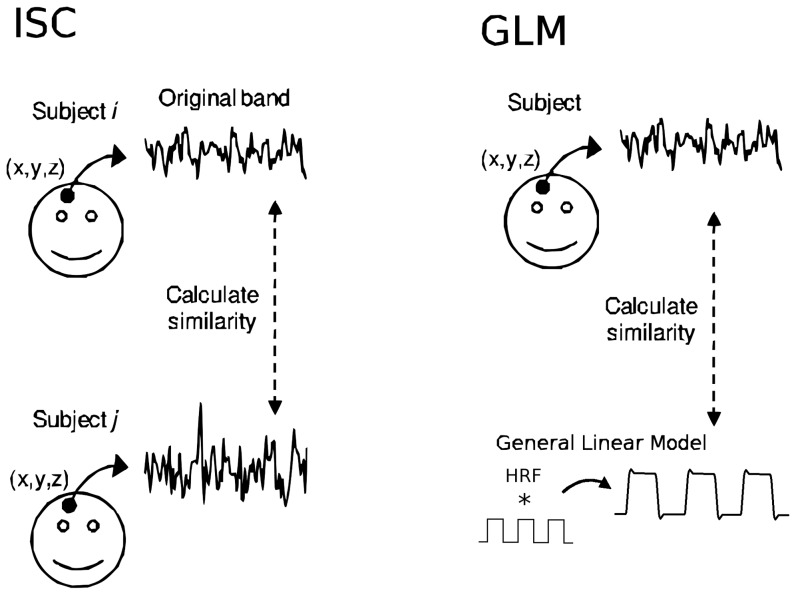
General conceptual difference between the non-parametric ISC and parametric GLM analysis. The ISCs are computed voxel-wise over the measured time series of every possible subject pair and then the results are combined to a single statistic. The GLM analysis fits the mathematical model (Here: boxcar function convolved with the canonical hemodynamic response function (HRF)) to the measured time-series of every subject and the group level results are then combined from the results of the individual subjects' analyses.

We use the GLM as a reference method in the comparison since it is a standard data analysis tool for locating brain activations in fMRI. The key difference between ISC and GLM methods is presented in Figure 1. ISC analysis combines voxel-wise correlations between several subject pairs in a fully non-parametric way to a single multi-subject statistical measure. Instead, GLM first compares voxel-wise the fMRI time series of each individual with a predefined model of the hemodynamic activity and then combines the results to a single multi-subject statistic. It is obvious that unlike the ISC method, where the model is not needed, GLM is not easily applicable to analyzing fMRI datasets acquired under complex stimuli for which the construction of the parametric model is far too difficult. Thus, it is necessary to use fMRI datasets which are acquired under strictly controlled experimental settings in order to carry out reliable validation, where the parametric model is guaranteed to succeed extremely well and this way provide the ground-truth for the non-parametric study.

## Materials and Methods

### ICBM functional reference battery data

For this study, we used fMRI data from the measurements with Functional Reference Battery tasks developed by the International Consortium for Human Brain Mapping (ICBM) [13] (http://www.loni.ucla.edu/ICBM/Downloads/Downloads_FRB.shtml) The data was obtained from ICBM database in the Image Data Archieve (IDA) of the Laboratory of Neuro Imaging (LONI) (http://www.loni.ucla.edu/ICBM). The ICBM project (Principal Investigator John Mazziotta, M.D., University of California, Los Angeles) is supported by the National Institute of Biomedical Imaging and BioEngineering. ICBM is the result of efforts of co-investigators from UCLA, Montreal Neurologic Institute, University of Texas at San Antonio, and the Institute of Medicine, Juelich/Heinrich Heine University - Germany.

The selected data included measurements from 37 healthy right-handed subjects (19 men and 18 women; average age was 28.2 years from the range of 20–36 years), who had all performed the five selected tasks from FRB. The functional data was collected with a 3 Tesla Siemens Allegra fMRI scanner and the anatomical T1 weighted MRI data with an 1.5 Tesla Siemens Sonata scanner. The TR/TE times for the functional data were 4 s/32 ms, flip angle 90 degree, pixel spacing 2 mm and slice thickness 2 mm. The parameters for the anatomical T1 data were 1.1 s/4.38 ms, 15 degree, 1 mm and 1 mm, correspondingly.

Similarly to Bellec et al. [14], we restricted the age range of the subjects to 20–38 years. In the database, this resulted to 41 right-handed subjects who had fMRI measurements from all five different FRB tasks: auditory naming (AN), external ordering (EO), hand imitation (HA), oculomotor (OM) and verbal generation (VG). The image data was pre-screened before analysis to ensure high quality of the data. According to pre-screening, fMRI data from four subjects were discarded because of a poor data quality for at least one task in the battery.

#### FRB tasks

The detailed task definitions of the functional reference battery are included in the FRB software package and they are next explained briefly here. All the five FRB task designs had the same block-structure in their implementations and they consisted of consecutive ‘off’ and ‘on’ blocks. There were 12 blocks per run (6 ‘off-on’) and 3 volumes at the beginning of the run to wait for magnetisation stabilisation. The blocks lasted 28 s so that ‘off-on’ phases lasted totally 56 s. This created finally 5 min 48 s duration for the whole experiment where there were 12 blocks (six ‘off’ and six ‘on’ blocks) for each run with 7 volumes in each block.

In every task, the ‘off’ block instruction was the same: the subjects had to respond with the left mouse button press every time they saw an arrow pointing to the left. The different ‘on’ blocks were defined separately for each task.

In the first task, AN, subjects were instructed to listen to the description of an object from a sound file and then think their answer silently to the description. The stimulus had first 2 s of silence, then 1.5 s of description and finally again 2 s of silence. This is a language task with an auditory input modality and the FRB definition noted that auditory cortex should be activated here (in addition to language areas).

In the EO task, which is a working memory task, the subjects were presented with four abstract design stimuli followed by a fifth stimulus and required to recall whether the final abstract design was among the four presented previously. The designs were visible for 450 ms and the screen was blank 50 ms between the designs. The subjects responded via a button press whether the final stimulus was among the four previously shown. This test was repeated five times during each ‘on’ block.

In the HA task subjects were instructed to imitate the presented hand configuration with their right hand. The example hand configurations were presented to them with pictures on the screen. Each hand position was presented for 3.5 s. This is a task requiring higher order motor coordination and motor planning and in the FRB description, it was noted that this task should activate the frontal and parietal areas.

In the OM task subjects were watching an image including a central cross in the middle surrounded by 10 black boxes. Subjects were instructed to concentrate on the central cross and saccade to the surrounding box if it changed white for a moment. After this, they should have returned their gaze immediately to the central cross. In each ‘on’ block there were 20 fixation trials and 20 target trials. There were four fixations of each of the following durations: 800 ms, 1000 ms, 1200 ms, 1400 ms, and 1600. These were randomized and each were followed by a 200 ms target trial. This way the task was supposed to activate the visual system and the occipital lobe.

Finally, in the VG task, the images of certain objects were shown to the subjects on the screen and subjects were instructed to generate a verb associated to the object silently in their mind without saying it aloud. During the ‘on’ blocks, line drawings were presented for 0.5 s. This task is a language task with visual input and was noted to activate the language and visual areas.

#### Pre-processing

Pre-processing and the GLM part of statistical analysis were performed by using the program FSL (version 4.1.6) [Oxford Centre for Functional Magnetic Resonance Imaging of the Brain (FMRIB), Oxford University, Oxford, U.K.] [15]. The data processing in FEAT (version 5.98) was done in three phases. First, motion correction was performed using the FSL's MCFLIRT by maximizing the correlation ratio between each time point and the middle volume, using linear interpolation [16], [17]. Second, the Brain extraction tool (BET) [18] was applied to to extract the brain volume from functional data. Finally, the images were temporally high-pass filtered with a cutoff period of 60 s and the spatial smoothing was applied with a Gaussian kernel with full width at half maximum (FWHM) of 5 mm. The original data had 87 volumes with three stabilization volumes, which were discarded from the analysis. The brain extraction from the anatomical T1 images was also performed by BET, but this was done manually for each T1-weighted image separately from the FEAT procedure as the parameters of BET required individual tuning.

The image registration was performed in two phases using FSL Linear Registration Tool (FLIRT) [16], [17]. First, the skull-stripped functional images were aligned (6 degrees of freedom, full search) to the skull-stripped high-resolution T1-weighted image of the same subject, and then the results were aligned to the standard (brain only) ICBM-152 template (12 degrees of freedom, full search).

### Analysis Methods

#### General Linear Model with FEAT

After preprocessing, the GLM was performed at the single subject level with the FSL (FEAT, fMRI Expert Analysis Tool) [19], [20]. Most of the processing options were chosen according to the defaults of FEAT. The model was defined for 84 volumes where each block had the length of seven volumes. The length of the block in volumes was computed from the timing of the tasks and the scanning parameters (28 s divided by 4 s). The boxcar model was designed with the three-column format of FEAT. In this format it was possible to define separately for every block the current value of the model (one for each ‘on’ block), starting point in time from the beginning of experiment, and duration of the current block from the starting point. Then, the model was convolved with the canonical hemodynamic response function (HRF) (a single 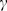-function modeling: phase 0 s, standard deviation 3 s, mean lag 6 s) along to its temporal derivative. Finally, the same default high pass filtering as applied to experimental data (with a cutoff of 60 s) was applied to the model. The analysis itself was performed with the FILM prewhitening procedure [21].

Higher-level mixed effects group analyses were performed for each contrast by using FSL's FLAME (FMRIB's Local Analysis of Mixed Effects) module with two stages (1+2), where the second stage estimation was performed using MH MCMC (Metropolis-Hastings Markov Chain Monte Carlo) sampling [19]. Voxel-wise False Discovery Rate (FDR) based multiple comparison correction [22], [23] under the independence or positive dependence assumption was used to threshold the z-statistic volumes. As argued in [9], the FDR based multiple comparison correction is a natural option for ISC and for this reason also the GLM thresholds were corrected with the FDR method. The used thresholding levels were q
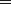
0.05, q
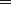
0.005, q
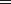
0.001 and the FDR corrected GLM thresholds are presented in the Table 1 for reference.

**Table 1:** FDR corrected GLM thresholds for different tasks.

	q 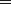 0,05	q 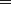 0,005	q 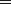 0,001
AN	0.0025	0.1483 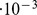	0.2186 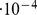
EO	0.0063	0.4530 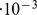	0.7259 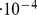
HA	0.0049	0.3574 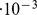	0.5872 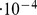
OM	0.0040	0.2723 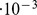	0.4233 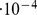
VG	0.0039	0.2626 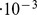	0.4104 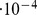
Average	0.0043	0.2987 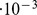	0.4832 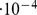

#### Inter-Subject Correlation Analysis

The ISC analysis was performed using ISCtoolbox for Matlab by Kauppi et al. [9] ( http://code.google.com/p/isc-toolbox/). This implementation can perform the ISC analysis over the specific frequency bands of the time series and threshold the results via voxel-wise resampling with the selected significance level. In this study, the analysis was performed only across the full frequency band.

In [9], the ISC is defined as a multi-subject similarity measure as follows. First, Pearson's correlation coefficient is calculated voxel-wise between every pair of subjects as:


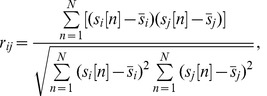
(1)

where 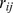 is the sample correlation coefficient between the time series, N is the total number of samples in time series, 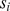 and 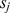 are time series obtained from the 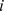
th and 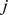
th subject, respectively, and 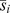 and 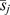 denote the means of 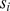 and 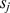.

To obtain the final multi-subject measure, the 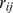 values from all subject pairs were combined into a single ISC statistic by averaging:


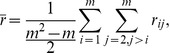
(2)

where 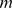 is the number of subjects. Since 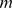 was 37 in our study, the correlation coefficients were averaged from 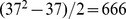 subject pairs.

The statistical inference with this measure is complicated by the dependency of 666 correlation coefficients. To account for this problem a fully non-parametric voxel-wise resampling test is implemented in the ISC toolbox. This test accounts for temporal correlations inherent to fMRI data (for details of the test, see [9]). Similar to [9], we approximated resampling distribution with 1,000,000 realizations and corrected the resulting p-values using an FDR-based multiple comparison correction with independence or positive dependence assumption [22], [23].

### Simulated Data

In order to obtain quantitative validation results against a known ground truth, we generated four sets of simulated imaging data with different noise levels mimicking the real data which was used in the study. Each set contained 37 simulated functional images in the standard ICBM-152 space. Each voxel in these images was either activated or not activated. Activation regions were selected according to the binarized GLM analysis results of AN task with the threshold level of q
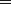
0.05. A hemodynamic signal was included in the timeseries of the voxels in the activated regions. The signal was selected to be exactly the same which was used as a model in the GLM analysis, i.e., a boxcar convolved with a canonical HRF. Finally, pink 1 f noise generated as described in [24] (https://ccrma.stanford.edu/~jos/sasp/Example_Synthesis_1_F_Noise.html) was added to every timeseries in the volume. The power of the noise was 100, 200, 500 and 1000 times stronger than the power of the included hemodynamic signal resulting to signal to noise ratios (SNR) of 0.01, 0.005, 0.002 and 0.0001. The areas outside the activated regions contained only the noise signal. The simulation procedure was exactly the same for every 37 simulated images, that is, we ignored the anatomical and effect size variations between subjects.

The pink noise was chosen in the simulations due to empirical evidence that fMRI noise time-series contains 1 f-like noise [25]. As the data was generated directly in MNI-152 coordinates no registration or motion correction was needed for the simulated data and pre-processing included only temporal and spatial filtering which were performed exactly as described for FRB data.

### Method comparison

We compared the results of the ISC analysis and GLM with two performance measures. The first measure was suitable for comparing non-thresholded statistical images and was based on Pearson's correlation coefficient:


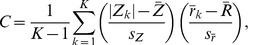
(3)

Here 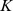 is the total number of brain voxels in the image (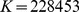 voxels) and 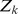, 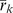 are the GLM and ISC statistics of the 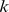
th voxel, respectively. The absolute value of the 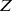 statistic was taken before computing the correlation measure because it was expected that both large negative and large positive 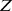-values relate to high 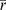 values. The 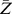 and 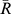 are the corresponding sample means and 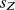, 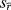 the corresponding standard deviations (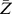 and 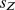 are computed from 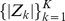).

Our second performance measure was the Dice index [26] which was suitable for comparing thresholded and binarized GLM and ISC maps. The binarized maps were created by assigning the value of one to a voxel if the statistic value passed the threshold and otherwise assigning the value of zero to it. Let 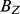 denote the set of activated voxels of GLM and 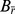 the set of those of the ISC. The Dice index between two sets was defined as:


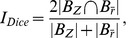
(4)

where the numerator measures the size of common activation occurrence and the denominator measures the sizes of activated areas according to individual methods. In other words, the equation measures the areas where both binaries are true against the areas where at least one binary is true. In practice the Dice index was computed from the binary vectors. The thresholded and binarized statistic volumes of the GLM and ISC analyses were vectorized by reshaping them to M-dimensional vectors. Then, the Dice index was computed as follows:


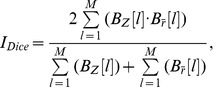
(5)

where 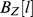 and 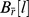 are the 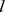
th voxels of binary vectors reshaped from binarized GLM and ISC statistic volumes, respectively. The sums were computed over the whole volumes (M = 91×109×91 = 902629 voxels).

The resulting Dice index values vary between 0–1, where 1 denotes the exact similarity and 0 denotes no overlap. To further ease the interpretation of Dice indices, we can utilize the relationship between the Dice index and Kappa coefficient. Zijdenbos et al. showed that under certain assumptions [27], which are valid here, the Dice index is (asymptotically) equal to Kappa coefficient. According to Landis et al. [28] the Kappa coefficient values can be divided into six categories: less than 0, “No agreement”; 0–0.2, “Slight agreement”; 0.2–0.4, “Fair agreement”; 0.4–0.6, “Moderate agreement”; 0.6–0.8, “Substantial agreement”; 0.8–1.0, “Almost perfect agreement”. These categories are ad-hoc, but widely used. The relationship between Dice index and Kappa coefficient is further described by Finch [29]. Dice index was chosen instead of Kappa, because it is better suited to for our purposes since it ignores the non-activated regions (see [27] for more details) and it is widely used as the performance index in the evaluation of medical image segmentation algorithms.

## Results

Pearson's correlations, Eq. (3), between the absolute values of the 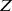-statistic of GLM and ISC are presented in Table 2. The values of the correlation coefficients were between 0.69 and 0.83, where the lowest correlation was from the task EO and the highest from the task HA. The average of the correlation coefficients across all of the tasks was 0.74. These values indicate a high similarity between the test statistics of GLM and ISC.

**Table 2:** Voxel-wise correlation measures, Eq. (3).

TASK	AN	EO	HA	OM	VG	Average
C	0,69	0,69	0,83	0,76	0,75	0.74

**Figure 2:**
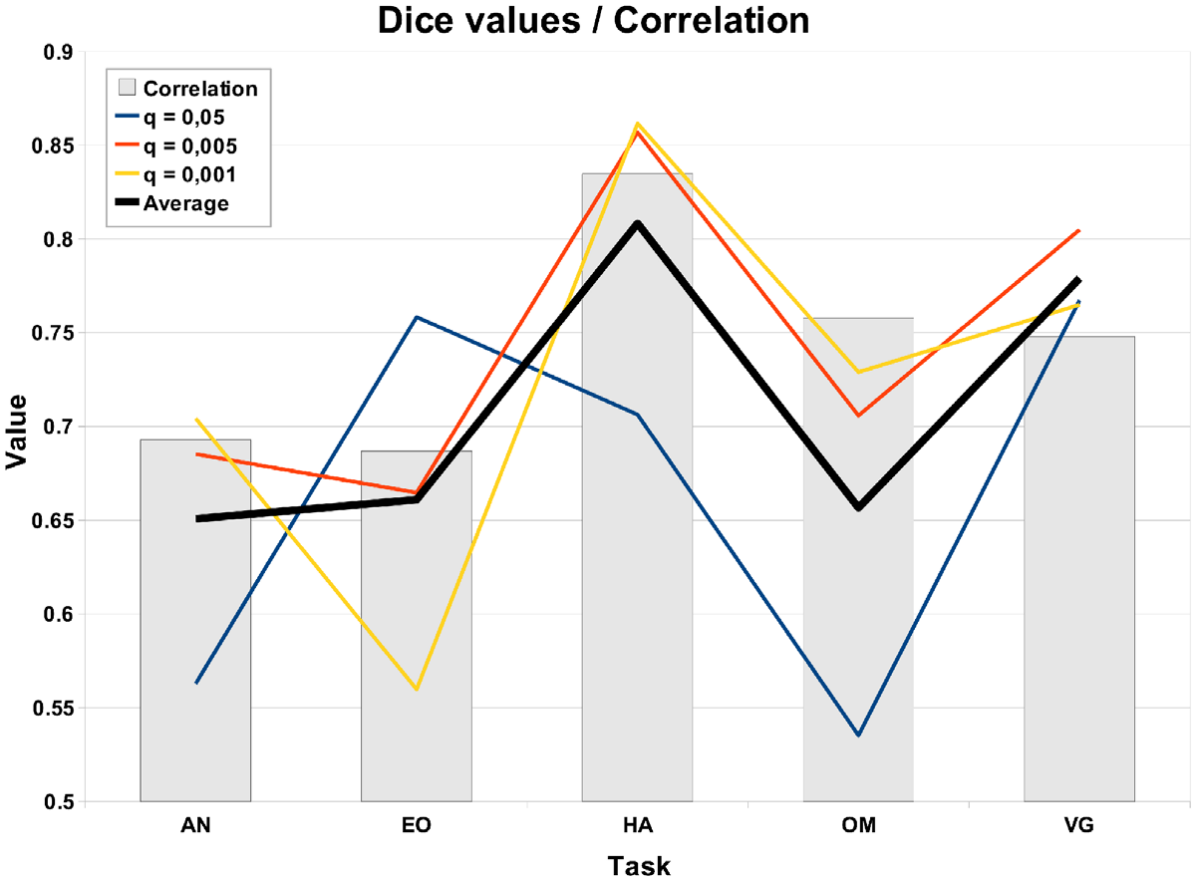
The correlation measure and the Dice index. The bars show the correlation measure between ISC and GLM and the lines present the Dice index values from different significance levels. The continuous black line presents the average over the Dice values within the current task. The HA task has higher correlation measure than other tasks and a high Dice index value. The EO task has the lowest correlation measure and the Dice index is also lower and varies the most with the thresholds. This suggests that a high correlation measure predicts a high Dice index value. We note that the values used as the basis for this figure are of higher numerical precision than those reported in Tables 2 and 3.

The Dice index, Eq. (4), between binary maps resulted in the average value of 0.73 across the tasks and the thresholds. The average Dice index values across the three thresholds for the specific tasks ranged from 0.65 to 0.81. The average over the tasks varied from 0.72 to 0.74 depending on the threshold. The results are presented in the Table 3. When comparing these with the Kappa categories discussed earlier, the similarity of the thresholded statistical maps of ISC and GLM had a moderate (0.4–0.6, 3 values), substantial (0.6–0.8, 9 values) or almost perfect (0.8–1.0, 3 values) agreement. Most of the Dice indices were at the level of substantial agreement. The Dice index values of the VG task were most stable across the thresholds (0.77, 0.81, 0.77), whereas the corresponding values of the EO task were most variable (0.76, 0.66, 0.56). With the tasks AN, HA, and OM, the values of the Dice indices with the two tightest threshold levels were close to each other but the values were notably lower with the most liberal level. These results indicated that the q = 0.05 level might be too liberal for this kind of study. The correlation and Dice index results are visualized together in Figure 2.

**Table 3:** Dice Indices, Eq. (4).

Task/Threshold	q 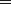 0.05	q 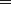 0.005	q 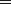 0.001	Average
AN	0.56	0.69	0.7	0.65
EO	0.76	0.66	0.56	0.66
HA	0.71	0.86	0.86	0.81
OM	0.54	0.71	0.73	0.66
VG	0.77	0.81	0.77	0.78
Average	0.72	0.74	0.72	0.73

According to Landis et al. [28] the results can be categorized as following: less than 0, “No agreement”; 0–0.2, “Slight agreement”; 0.2–0.4, “Fair agreement”; 0.4–0.6, “Moderate agreement”; 0.6–0.8, “Substantial agreement”; 0.8–1.0, “Almost perfect agreement”. By comparing the results with these categories the HA task can be nominated to have “Almost Perfect” agreement and the EO task, which had the lowest results as “Substantial agreement” even it also has values from “Moderate agreement” level.

**Figure 3:**
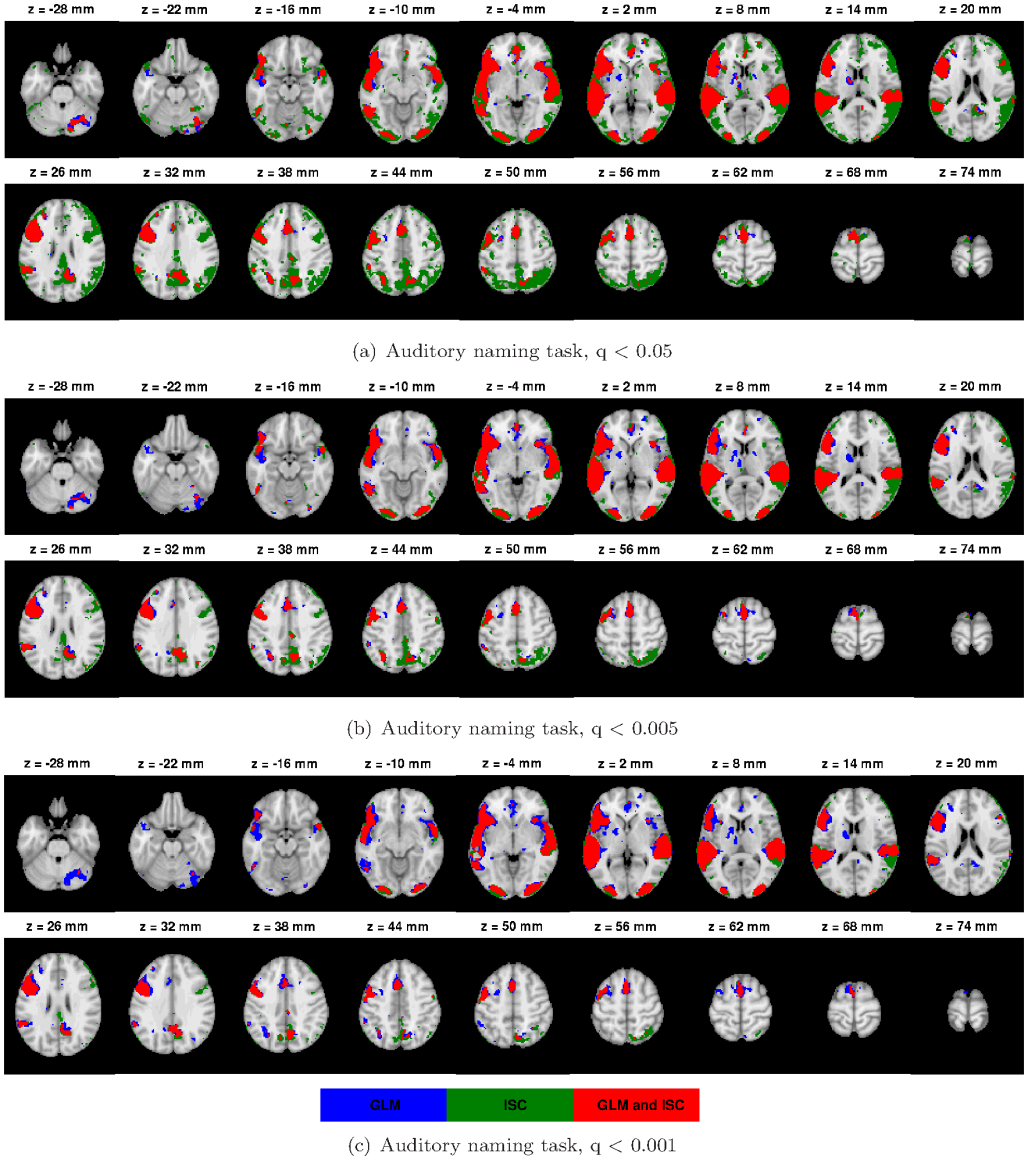
GLM and ISC analysis results for the AN task (thresholded and FDR corrected, q 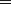
 **0.05 (a), q** 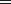 **0.005 (b), q** 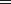 **0.001 (c) ).** In the images, the red color indicates voxels which are activated according to both ISC and GLM methods, blue indicates voxels activated according to GLM but not according to ISC and green indicates voxels activated according to ISC but not with GLM. The images are in neurological orientation. There is a notable correspondence between the ISC and GLM maps especially in auditory cortex, visual cortex, and cingulate gyrus. We can also see that the ISC analysis was clearly more liberal than the GLM analysis with a loose threshold (q
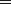
0.05), but became more conservative when the thresholds became tighter (q
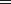
0.005 and q
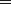
0.001).

**Figure 4:**
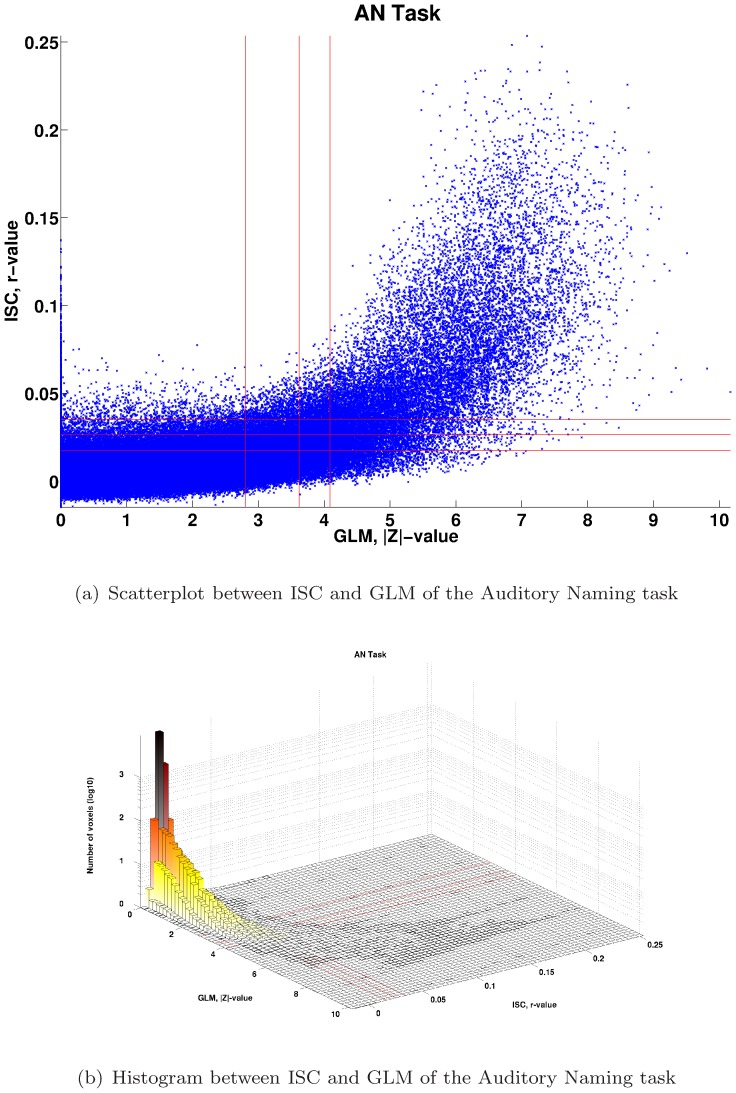
GLM and ISC analysis results for the AN task. The scatterplot (a) presents the voxel-wise statistic values of GLM (horisontal axis) and ISC (vertical axis). Red lines define the thresholds with levels q = 0.05, q = 0.005 and q = 0.001. The second image (b) displays the corresponding histogram, which shows more clearly how the mass of the values is distributed with respect to the thresholds defined by the red lines. Most of the values are focused close to the origin which is not visible in the scatterplot.

**Figure 5:**
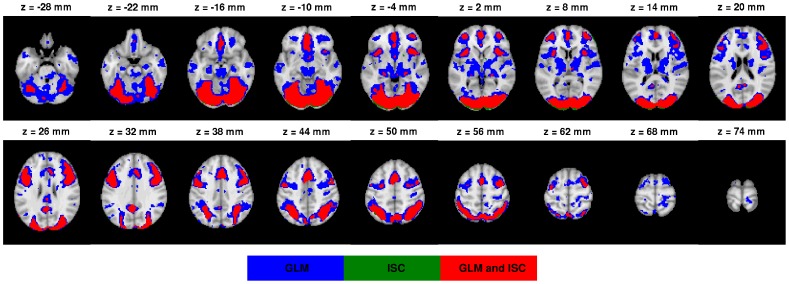
GLM and ISC analysis results for the EO task. In the image the thresholded (FDR corrected, q
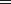
0.001) results for EO task are presented as a binary overlay image. The color coding in the image is the same as in Figure 3. The threshold images from the levels q
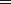
0.05 and q
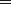
0.005 are visible in the Figure S1 of the Supplement. Both methods find the same activation areas widely across the brain, including lateral occipital cortex, inferior frontal gyrus, precentral gyrus and supplementary motor cortex. Note also how ISC only (green) and commonly detected areas (red) are vanishing faster than GLM only areas (blue) when the threshold becomes more conservative. Thus, the ISC analysis was more conservative of the two methods especially with the lowest q-value. This tendency explains relatively high variation in the Dice index values with different significance levels for this particular task.

**Figure 6:**
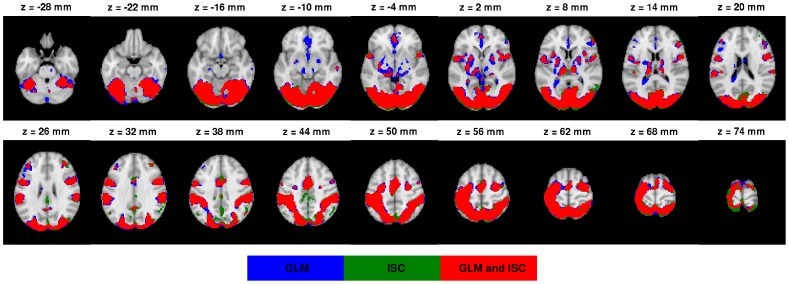
GLM and ISC analysis results for the HA task. In the image the thresholded (FDR corrected, q
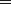
0.001) results for HA task are presented as a binary overlay image. The threshold images from the levels q
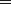
0.05 and q
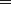
0.005 are visible in the Figure S3 of the Supplement. The color coding in the image is the same as in Figure 3. Here it is clear that commonly detected areas (red) are dominant. There are also a notable number of ISC only detections (green), which might indicate that ISC can detect activations which are not detectable by GLM. On the other hand, some GLM only activations were located in cerebrospinal fluid, which suggested that there might exist measurement artifacts.

The Figure 3 presents all three threshold levels q
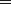
0.05 (a), q
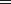
0.005 (b), and q
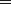
0.001 (c) of the AN task. The Figure 4 (a) presents a voxel-wise scatter plot between GLM (horisontal axis) and ISC values (vertical axis). Figure 4 (b) presents the corresponding histogram, which shows more clearly how the mass of the values is distributed with respect to the thresholds. The red lines in the Figure 4 denotes the three thresholds. The scatterplots and histograms of the other tasks are present in the Figures S2, S4, S6 and S8 of Supplement. The thresholded statistical maps of GLM and ISC with the threshold level q
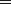
0.001 are presented in Figure 3 (c) for AN task and Figures 5 and 6 for EO and HA tasks. The threshold images from tasks EO and HA with threshold levels q
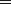
0.05 and q
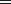
0.005 are presented in Figures S1 and S3 of Supplement. Similarly to Figure 3, the Figures S5 and S7 of Supplement presents all three threshold levels for the tasks OM and VG respectively. In the figures, the red color indicates those voxels, which are activated according to both methods, the blue color indicates activated voxels according to GLM analysis only and the green color denotes activated voxels according to ISC analysis only. The images are in neurological orientation.

With the AN task, both methods detected activations in auditory cortex, visual cortex, and cingulate gyrus (see Figure 3). This was as expected based on the FRB task definition and comparison to the previous fMRI studies with the AN task through a meta-analysis tool Pubbrain (http://www.pubbrain.org). With the EO task, the activations according to both methods were in lateral occipital cortex, inferior frontal gyrus, precentral gyrus and supplementary motor cortex (see Figure 5). As we expected, these results were highly similar to the detected activations of the healthy control subjects in the study of Hamilton et al. [30] which studied the same EO task as we were using here. With the HA task, there were activations in multiple parietal areas and inferior frontal gyrus and cingulate gyrus in the frontal lobe (see Figure 6). These were as expected (the FRB description noted that this task should activate at least frontal and parietal areas). With the HA task, ISC (but not GLM) detected activation in precuneous cortex. The activation remained visible even with the tightest threshold presented in Figure 6. Based on a review [31], it seems plausible that the precuneous is active during the hand imitation task. With the OM task, there were activations present at precentral gyrus, occipital pole, supplementary motor cortex and lateral occipital cortex (see Figure S5 of Supplement). These were as expected as the FRB description noted that the task should activate the visual system and the occipital lobe. With the VG task, activations at inferior temporal gyrus, inferior frontal gyrus, temporal occipital fusiform cortex, lingual gyrus, occipital pole, lateral occipital cortex and supplementary motor cortex were detected (see Figure S7 of Supplement). These were as expected as the FRB definition noted that the task should activate language and visual areas.

**Figure 7:**
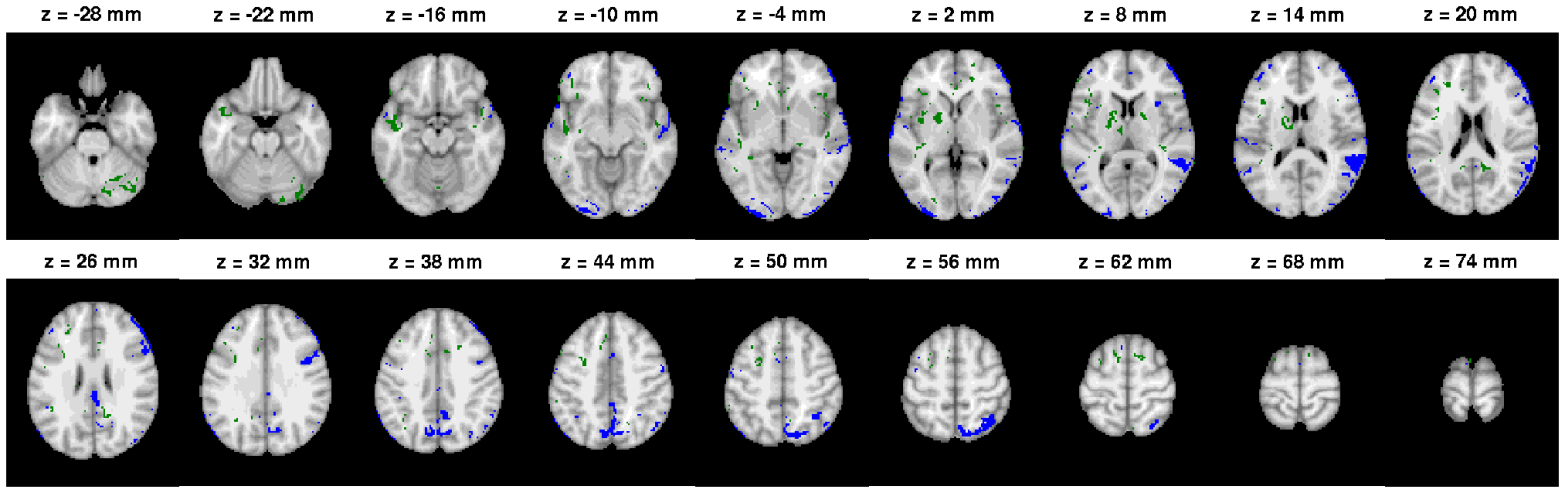
The voxels consistently detected as activated by one method and not by the other with AN task. Green color indicates voxels which were detected as activated by GLM in all thresholding levels, but not detected as activated by ISC in even the most liberal thresholding level (q = 0.05). Viceversa, blue color indicates voxels which were detected as activated by ISC in all of the thresholding levels, but not detected as activated by GLM with even the most liberal thresholding level (q = 0.05). Mostly these are isolated voxels or voxels lying near the boundary of the activation area. However, the ISC detected activations in Posterior and Anterior cingulate cortex and Precuneus as well as Occipital lobe that were not detected by the GLM. These areas are suspected to overlap with the default mode network in several studies, e.g., [34]–[36].

Two general trends were noticeable from the overlay images. First, with the EO (Figure 5) and VG tasks (Figure S7 of Supplement), the ISC analysis was generally more conservative than the GLM analysis for detecting activation areas, because the number of voxels detected only by GLM (blue) was high and common areas (red) were surrounded by these (blue) areas. Second, with the tasks AN, HA and OM, ISC tended to find more activated voxels than the GLM when the most liberal threshold (q
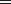
0.05) was used. Thus, GLM analysis was more conservative of the two methods. However, the situation was reversed when the most tightest threshold (q
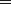
0.001) was used, i.e., ISC analysis became more conservative than the GLM analysis. This is also visible in the Figure 7, which presents the voxels that were consistently detected as activated up by one method and not the other method for the AN task. Corresponding images for other tasks are presented in Figures S9, S10, S11 and S12 of Supplement.

The correlation measure was computed between 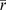-statistics and 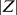-statistics instead of signed Z-statistics. This was done because it was expected that both high negative (de-activations) and high positive (activations) 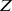-values relate to high positive 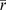 values. To validate this hypothesis, we computed the correlation between signed 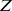-values and 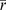-values. In that case, the correlation measures dropped to 0.50, 0.53, 0.74, 0.70 and 0.57 for AN, EO, HA, OM and VG tasks, respectively. By comparing these values to the values in Table 2, we can see that the decrease was larger with the low correlation tasks (0.19 (AN), 0.16(EO) and 0.18 (VG)) and smaller with high correlation tasks (0.10 (HA), 0.06(OM)).

**Figure 8:**
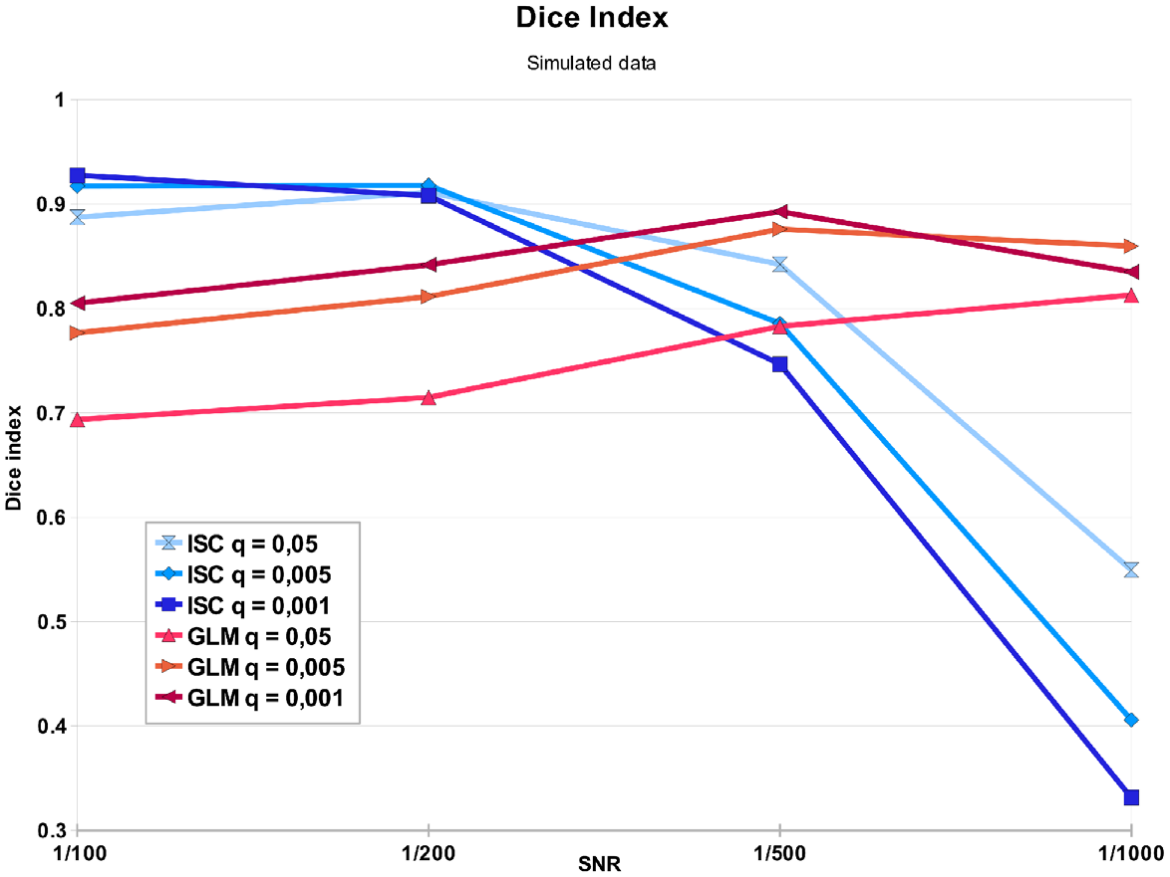
Similarity of the detected activation region and ground truth activation region in the simulation study. The lines present the Dice index values between the simulated versus detected activation area by ISC with different thresholding levels (blue lines) and by GLM with different thresholding levels (red lines). The ISC performed well with lower noise levels (SNR 1/100 and 1/200) but failed with the highest noise level (SNR 1/1000). The GLM performed overall well, but has a lower detection rate at low noise levels compared to ISC. This is due to false positive detections on the areas nearby ground-truth activation areas due to the effects of the spatial smoothing.

With the simulated data, the Dice indices between the activations detected (either by ISC or GLM) and the ground truth are presented in the Figure 8 for different noise and thresholding levels. Average Dice index was 0.76 for ISC and 0.81 for GLM. The non-parametric ISC method detected simulated activations very accurately when the SNR was 0.002 or greater. Only with the highest noise level and especially with the most conservative thresholding level, the accuracy of ISC was poor (Dice index below 0.4) as it failed to detect the truly activated voxels. The lower Dice indices for GLM with the two lowest levels of noise were due to enlargening of the activation regions due to filtering. In other words, the GLM-based analysis was too sensitive in this highly idealized setting. Overall, we consider that the performance of the two methods was similar at the three lowest noise levels and only at the highest noise level the advantages of using stimulus model derived information as in GLM became clearly apparent.

## Discussion

We have compared activations detected by two different fMRI data analysis methods: a standard model-based GLM method and a non-parametric ISC method. The major difference between these two flavours of analyses is that the the former requires a model for the stimulus time course while the latter is completely non-parametric in the sense it does not require any parametric form for the stimulus time-course. This means that the ISC can be used to analyze fMRI data acquired from the experiments of complex multi-dimensional stimuli, e.g., a movie. The used datasets were deliberately chosen so that they were optimized for the GLM type analysis to maximize the accuracy of the GLM analysis. The data was acquired from the ICBM research database, which contains fMRI acquisitions during highly standardized FRB stimuli. The data was pre-processed and separately analyzed with GLM (FSL) and ISC [9]. The Pearson's correlation was computed between corresponding statistics of ISC and GLM. The statistical maps from both methods were thresholded while accounting for the multiple comparisons based on FDR. The resulting binarized thresholded maps were compared by computing Dice index between them.

The correlations between GLM and ISC statistics validated the original assumption of the similarity of the measures used to quantify the activations. The average correlation value over all five tasks was 0.74, which can be considered as a high correlation. The average Dice-index over all five tasks varied between 0.72 and 0.74 depending on the task. As noted earlier, nine of the 15 Dice values were classified as substantial agreement (0.6–0.8) and three of the 15 as almost perfect agreement according to a widely used Landis and Koch categorization. Not surprisingly, the tasks with the highest Pearson's correlations featured the highest (and the most stable) Dice index values.

Accordingly, the activations detected by ISC matched well with the activations detected by GLM. The activation maps presented in Figure 3 and Figures 5 and 6 illustrate that ISC method was slightly more conservative than GLM method especially at the most conservative thresholding level q
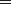
0.001 presented in the figures. The development is easiest to see from the Figure 3 where all threshold levels are present (See also the Figures S5 and S7 of Supplement). In most of these cases, the area of common activation (in red) was surrounded by GLM only activation area (in blue) indicating that ISC had found the same overall activation location as GLM method. This result is promising from the fMRI data analysis point of view under naturalistic paradigms, because it suggests that the nonparametric ISC method can locate true sources of BOLD signal activity well and yet it is not susceptible to spurious findings, easily leading to overinterpretation of the results.

The variation in the correlation measure (range 0.69–0.83) and Dice index (range 0.54–0.86) could have resulted from the differences in the nature of the behavioral tasks. Especially, the EO task had lower correlation value and Dice index than other tasks, probably because it is the most complex task in FRB designed to activate working memory. Surprisingly, the Dice and correlation measures of the AN and the VG tasks were different although the tasks are similar.

The simulation study demonstrated that the ISC could in principle accurately detect activations even when the signal to noise ratio was as low as 0.002. The lower Dice index values of GLM than those of ISC with the simulated databases with low noise levels (SNR 0.01 and 0.005) could be largely attributed to the spatial smoothing applied to the data before analysis. (With higher noise levels, the leakage of the activation to the voxels surrounding the true activation region by smoothing became harder to detect and thus GLM detected more accurately true activation areas.) As the FWHM of the smoothing kernel was the same for both methods this indicates ISC was more conservative (or less sensitive) than GLM. This phenomenon was observed also with experimental data - albeit to a lesser extent. As the simulation model was idealized and greatly simplified ignoring all between-subject variability, the results with simulated data should be interpreted with caution expecially regarding the exact noise levels that ISC could tolerate.

In this study, we used a relatively large database of 37 subjects. One interesting topic for future research would be to test comprehensively how the number of subjects affects the ISC analysis and what is the minimum number of subjects for the ISC analysis. Some results in this direction were presented by Hanson et al. [12] who demonstrated (but did not quantify) the stability of Roy's largest root statistic based ISC analysis after six or more subjects with a video stimulus of length of 156 s. However, for example, the reproducibility of ISC across subject samples remains an almost untouched research topic. Another slightly unusual aspect of the data is rather long TR of 4 seconds. It is difficult to speculate what effects this would have to the results of the method comparison as the two methods might react differently to the shortening of repetition time. However, it is important to note that recent ISC applications have typically used shorter TRs from 1.5 to 2 seconds.

Certain methodological choices warrant commenting. The GLM was used as the reference method because it is the standard method for analyzing fMRI studies acquired under a strictly controlled stimulus. The particular implementation of the multi-subject GLM (FSL's FLAME using MCMC) was selected because it is widely used and properly evaluated [15], [20]. In particular, a computationally heavy MCMC approach was selected due to its accuracy [20]. Obviously, activations detected by GLM cannot be considered as ground truth and we therefore verified that our GLM analysis results to matched to the prior expectations based on fMRI literature. This was done by comparing our analysis results with the information available through a meta-analysis tool Pubbrain. In the GLM-based fMRI analysis, it is often recommendable and more typical to apply a family-wise error rate based multiple comparisons correction (either in voxel or cluster level) instead of a more liberal FDR-based criterion adopted by us (see [32] for a comparison of different multiple comparison options in fMRI). We adopted it, since FDR is a natural choice for ISC analysis and it is essential to compare detected activations at the same significance level. Indeed, as can be noted based on Figure 3, especially the FDR level q
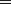
0.05 was liberal (technically, we could expect 5% of the activated voxels to be false positives) and some of the activations were likely to be due to imaging artefacts. In visual inspection, both ISC and GLM seemed to detect activations that could be suspected to be artefactual at the most liberal thresholding level while at the most conservative thresholding level activations that could be easily labeled as artefactual were almost non-existent.

Obviously, there are also methodological choices related to the ISC analysis although the methodological literature about ISC is scarce compared to that of the model-based GLM analysis. The first choice is that of the test statistic, in this work given in Eq. 2. Alternatives to this statistic include average of Z-transformed correlation coefficients [3], Roy's largest root [12], and average correlation coefficient between subjects response time-course and an averaged response time course [33]. In the latter, the order of the averaging and normalization to unit variance is reversed compared to our test statistic leading to a different (but related) test-statistic. Our preference of the test statistic selected in this work relate to its easy interpretation in the simple case that the true correlation between all subjects' time series has an equal value (see [9]). However, we speculate that the choice of test statistic is not critical unless the number of subjects or time-points is much smaller than here and, in particular, the qualitative results of this work do not rely on a particular choice of test statistic. The second, we think more critical, choice is that of the thresholding procedure. The important question here is if the hypothesis testing relying on parametric models (e.g. [3]) could replace more computationally heavy resampling procedures (e.g. [33], [9], and this work). In this work, we have experimentally shown that a time-domain resampling test produces inference results comparable to model-based activation detection. Further work is required to identify the most optimal thresholding scheme.

An interesting detail can be observed by studying activations detected only by ISC colored in blue in Figure 7. These activations detected by solely by ISC included voxels from Posterior and Anterior cingulate cortex and Precuneus as well as Occipital lobe. Similar patterns of activations detected solely by ISC can also be found by inspecting the Figures S10 and S11 in the Supplement. These areas are suspected to overlap with the default mode network in several studies, e.g., [34]–[36]. In a wider scope, [8] suggested that that naturalistic stimulation may provide a complementary tool to the resting state protocol for studying the default mode network.

Both the ISC- and GLM-based statistics presented here focus on shared responses across subjects while allowing some intersubject variablity in the models via mixed effects modelling (GLM) or how the hypothesis testing is performed (ISC). This seems to be a reasonable assumption in the tasks presented here, but under other kind of experiments intersubject variability can be considerably higher and harder to model due to individual differences in information processing. The investigation of these differences requires the use of more sensitive methods which take better into account the variability across subjects. For instance, clustering approach presented in [37] preserves the entire structure of the intersubject correlation matrices, making it a suitable method for investigating differences and similarities in brain responses in data-driven manner even for a large group of subjects simultaneously. Another approach was presented in [38], where individual differences were investigated by comparing the results of group-level ISC analysis and intra-subject correlation analysis computed across repeated presentations.

Our results indicate that the ISC analysis can be used to find the same activation areas as the stimulus model-based GLM analysis when the parametric form of the stimulus is known. The motivation for this study is that ISC-based methods do not require the model of the stimulus time course and therefore they can be used in many research settings where the parametric modeling of the stimulus is not applicable. For example, movies provide an interesting form of a more naturalistic stimulus that is impossible to model completely and where the applicability of the parametric model based methods for activation detection is therefore limited.

## Supporting Information

Figure S1
**GLM and ISC analysis results for the EO task.** In the image the thresholded (FDR corrected, q
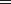
0.05 (a) and q
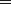
0.005 (b)) results for EO task are presented as a binary overlay image. The color coding in the images is the same as in Figure 3 of the article. The image of q
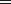
0.001 is presented in the Figure 5 of the article. Both methods find the same activation areas widely across the brain, including lateral occipital cortex, inferior frontal gyrus, precentral gyrus and supplementary motor cortex. Note also how ISC only (green) and commonly detected areas (red) are vanishing faster than GLM only areas (blue) when the threshold becomes more conservative. Thus, the ISC analysis was more conservative of the two methods especially with the lowest q-value. This tendency explains relatively high variation in the Dice index values with different significance levels for this particular task.(TIFF)

Figure S2 **GLM and ISC analysis results for the EO task.** The scatterplot (a) presents the voxel-wise statistic values of GLM (horisontal axis) and ISC (vertical axis). Red lines define the thresholds with levels q = 0.05, q = 0.005 and q = 0.001. The second image (b) displays the corresponding histogram, which shows more clearly how the mass of the values is distributed with respect to the thresholds defined by the red lines. Most of the values are focused close to the origin which is not visible in the scatterplot.
(TIFF)

Figure S3
**GLM and ISC analysis results for the HA task.** In the image the thresholded (FDR corrected, q
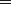
0.05 (a) and q
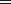
0.005 (b)) results for HA task are presented as a binary overlay image. The color coding in the images is the same as in Figure 3 of the article. The image of q
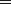
0.001 is presented in the Figure 6 of the article. Here it is clear that commonly detected areas (red) are dominant. There are also a notable number of ISC only detections (green), which might indicate that ISC can detect activations which are not detectable by GLM. On the other hand, some GLM only activations were located in cerebrospinal fluid, which suggested that there might exist measurement artifacts.(TIFF)

Figure S4 **GLM and ISC analysis results for the OM task.** The scatterplot (a) presents the voxel-wise statistic values of GLM (horisontal axis) and ISC (vertical axis). Red lines define the thresholds with levels q = 0.05, q = 0.005 and q = 0.001. The second image (b) displays the corresponding histogram, which shows more clearly how the mass of the values is distributed with respect to the thresholds defined by the red lines. Most of the values are focused close to the origin which is not visible in the scatterplot.
(TIFF)

Figure S5
**GLM and ISC analysis results for the OM task.** In the image the thresholded (FDR corrected, q
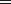
0.05 (a), q
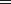
0.005 (b) and q
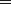
0.001 (c)) ) results for OM task are presented as a binary overlay image. The color coding in the images is the same as in Figure 3 of the article. As earlier with the HA task in Figure S3, also here ISC was first very liberal q
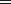
0.05 and there was mainly common (red) and ISC only (green) areas. When the threshold gets tighter q
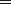
0.005 the ISC only areas becomes smaller like with AN task and with the tightest threshold q
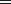
0.001 ISC becomes more conservative than GLM. Here some ISC only areas remained visible even with the tightest significance level q
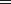
0.001.(TIFF)

Figure S6 **GLM and ISC analysis results for the OM task.** The scatterplot (a) presents the voxel-wise statistic values of GLM (horisontal axis) and ISC (vertical axis). Red lines define the thresholds with levels q = 0.05, q = 0.005 and q = 0.001. The second image (b) displays the corresponding histogram, which shows more clearly how the mass of the values is distributed with respect to the thresholds defined by the red lines. Most of the values are focused close to the origin which is not visible in the scatterplot.
(TIFF)

Figure S7
**GLM and ISC analysis results for the VG task.** In the image the thresholded (FDR corrected, q
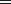
0.05 (a), q
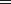
0.005 (b) and q
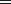
0.001 (c)) results for VG task are presented as a binary overlay image. The color coding in the images is the same as in Figure 3 of the article. Here we can see the similar progress than with the task EO. There were merely a few ISC only areas (green) without GLM areas next to them and most of the common (red) areas were surrounded by GLM only areas (blue). When the threshold tightened from q
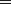
0.05 to q
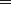
0.001 both ISC and GLM detections contracted, but ISC contracted somewhat faster, which again suggested that ISC was more conservative than GLM.(TIFF)

Figure S8 **GLM and ISC analysis results for the VG task.** The scatterplot (a) presents the voxel-wise statistic values of GLM (horisontal axis) and ISC (vertical axis). Red lines define the thresholds with levels q = 0.05, q = 0.005 and q = 0.001. The second image (b) displays the corresponding histogram, which shows more clearly how the mass of the values is distributed with respect to the thresholds defined by the red lines. Most of the values are focused close to the origin which is not visible in the scatterplot.
(TIFF)

Figure S9 **The voxels consistently detected as activated by one method and not by the other with EO task.** Green color indicates voxels which were detected as activated by GLM in all thresholding levels, but not detected as activated by ISC in even the most liberal thresholding level (q = 0.05). Viceversa, blue color indicates voxels which were detected as activated by ISC in all of the thresholding levels, but not detected as activated by GLM with even the most liberal thresholding level (q = 0.05). Mostly these are isolated voxels or voxels lying near the boundary of the activation area.
(TIFF)

Figure S10 **The voxels consistently detected as activated by one method and not by the other with HA task.** The color coding of the image is the same as in Figure S9. Mostly these are isolated voxels or voxels lying near the boundary of the activation area. However, the ISC detected activations in Precuneous cortex that were not detected by the GLM.
(TIFF)

Figure S11 **The voxels consistently detected as activated by one method and not by the other with OM task.** The color coding of the image is the same as in Figure S9. Mostly these are isolated voxels or voxels lying near the boundary of the activation area. However, the ISC detected activations in middle frontal gyrus that were not detected by the GLM.
(TIFF)

Figure S12 **The voxels consistently detected as activated by one method and not by the other with VG task.** The color coding of the image is the same as in Figure S9. Mostly these are isolated voxels or voxels lying near the boundary of the activation area. However, the ISC detected activations in middle temporal cortex and in superior cortex that were not detected by the GLM.
(TIFF)
