# Supplementary figures and images for: Inter-Subject Correlation in fMRI: Method Validation against Stimulus-Model Based Analysis
Source: PLoS One. 2012 Aug 8;7(8):e41196. doi: 10.1371/journal.pone.0041196 (PMC3414505; doi:10.1371/journal.pone.0041196)

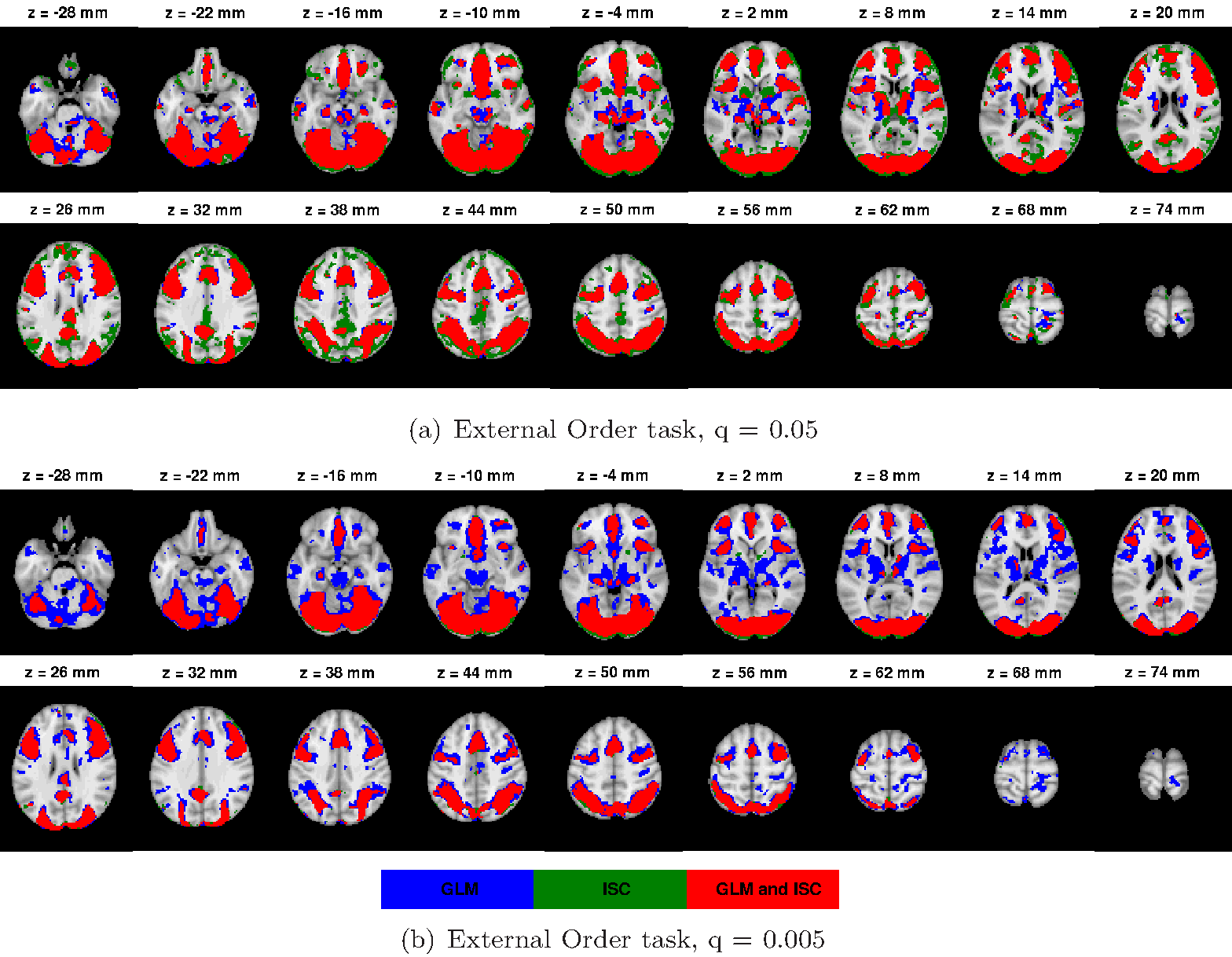

Supplement: Figure S1 — GLM and ISC analysis results for the EO task. In the image the thresholded (FDR corrected, q0.05 (a) and q0.005 (b)) results for EO task are presented as a binary overlay image. The color coding in the images is the same as in Figure 3 of the article. The image of q0.001 is presented in the Figure 5 of the article. Both methods find the same activation areas widely across the brain, including lateral occipital cortex, inferior frontal gyrus, precentral gyrus and supplementary motor cortex. Note also how ISC only (green) and commonly detected areas (red) are vanishing faster than GLM only areas (blue) when the threshold becomes more conservative. Thus, the ISC analysis was more conservative of the two methods especially with the lowest q-value. This tendency explains relatively high variation in the Dice index values with different significance levels for this particular task. (TIFF) [file pone.0041196.s001.tiff]

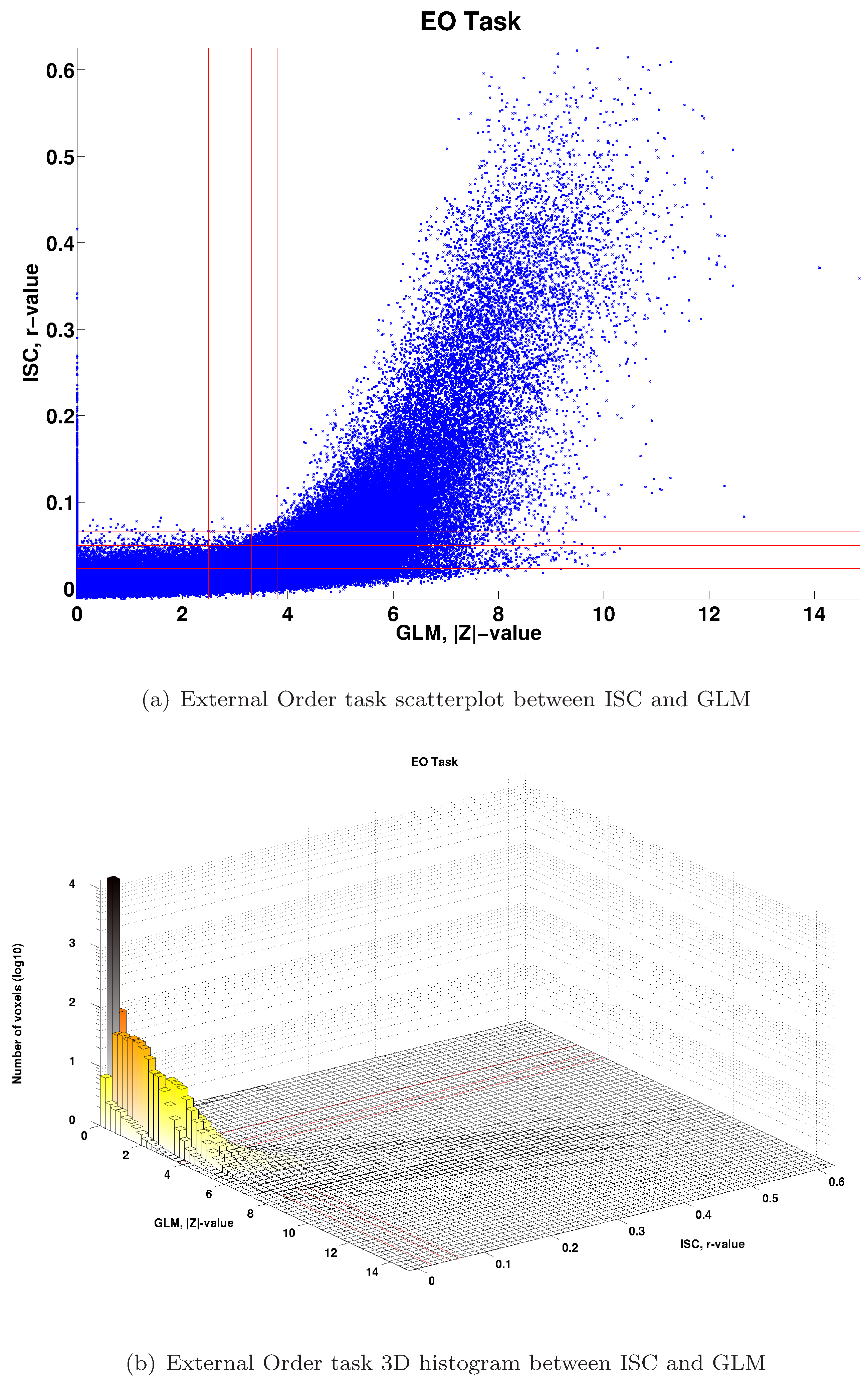

Supplement: Figure S2 — GLM and ISC analysis results for the EO task. The scatterplot (a) presents the voxel-wise statistic values of GLM (horisontal axis) and ISC (vertical axis). Red lines define the thresholds with levels q = 0.05, q = 0.005 and q = 0.001. The second image (b) displays the corresponding histogram, which shows more clearly how the mass of the values is distributed with respect to the thresholds defined by the red lines. Most of the values are focused close to the origin which is not visible in the scatterplot. (TIFF) [file pone.0041196.s002.tiff]

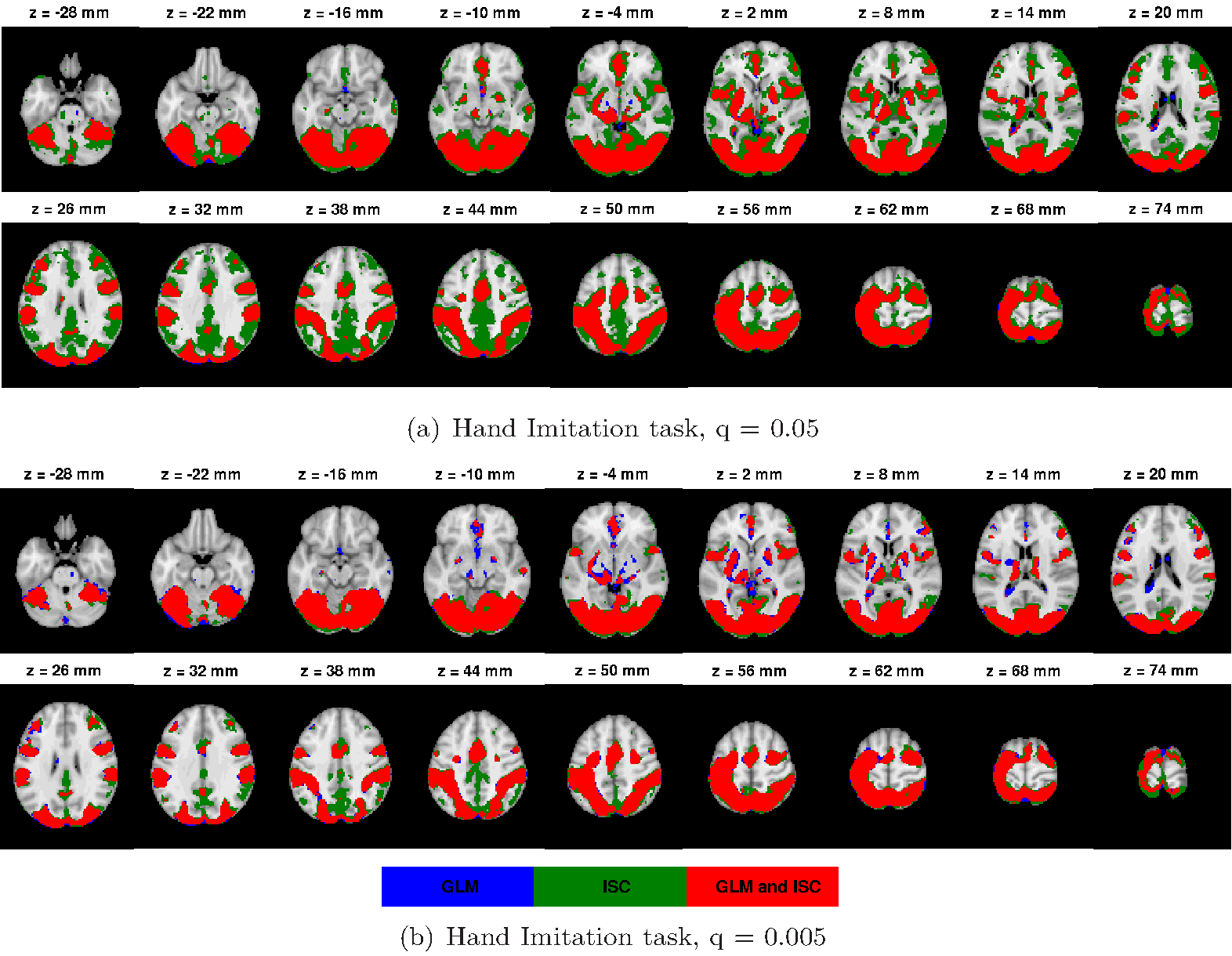

Supplement: Figure S3 — GLM and ISC analysis results for the HA task. In the image the thresholded (FDR corrected, q0.05 (a) and q0.005 (b)) results for HA task are presented as a binary overlay image. The color coding in the images is the same as in Figure 3 of the article. The image of q0.001 is presented in the Figure 6 of the article. Here it is clear that commonly detected areas (red) are dominant. There are also a notable number of ISC only detections (green), which might indicate that ISC can detect activations which are not detectable by GLM. On the other hand, some GLM only activations were located in cerebrospinal fluid, which suggested that there might exist measurement artifacts. (TIFF) [file pone.0041196.s003.tiff]

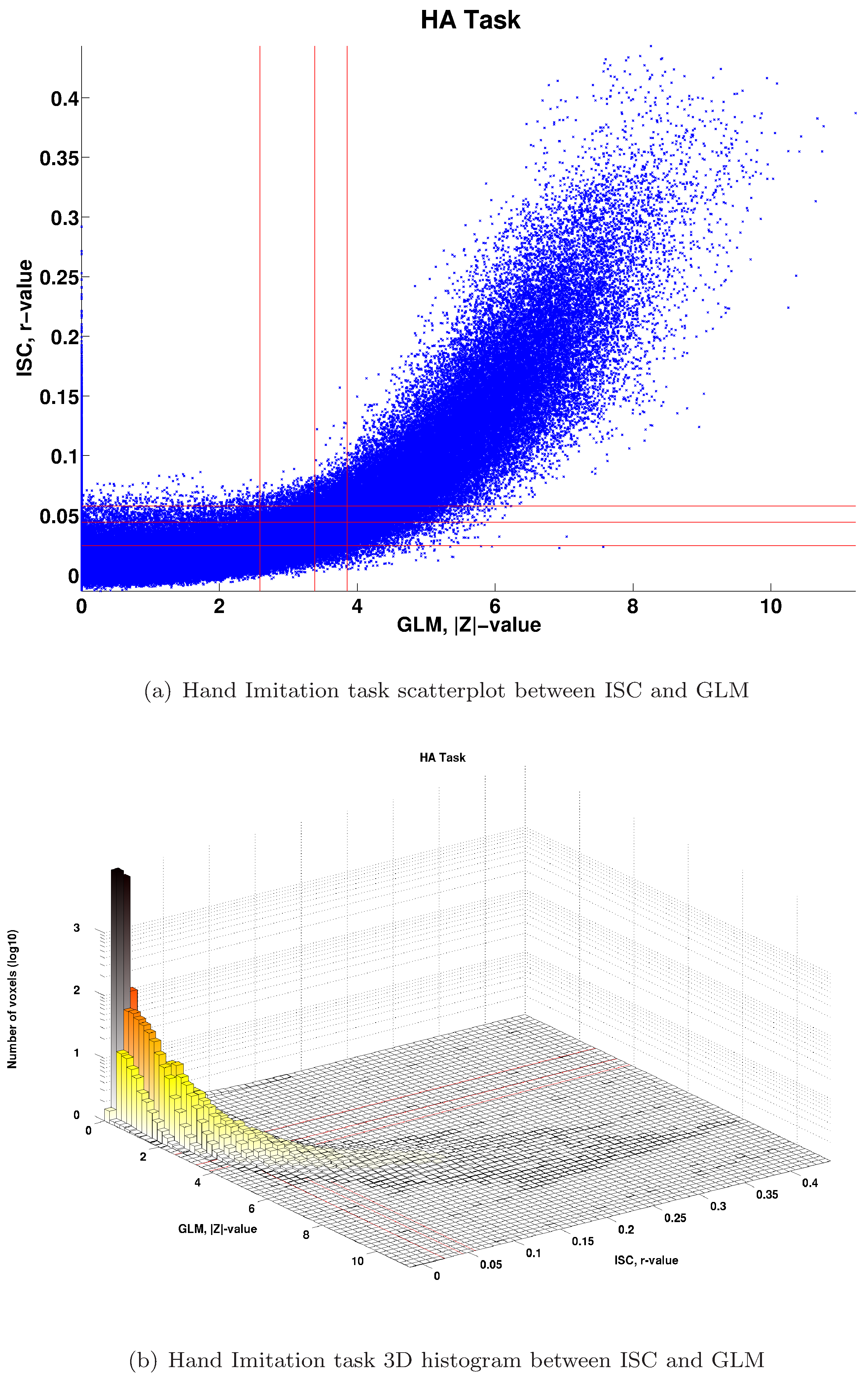

Supplement: Figure S4 — GLM and ISC analysis results for the OM task. The scatterplot (a) presents the voxel-wise statistic values of GLM (horisontal axis) and ISC (vertical axis). Red lines define the thresholds with levels q = 0.05, q = 0.005 and q = 0.001. The second image (b) displays the corresponding histogram, which shows more clearly how the mass of the values is distributed with respect to the thresholds defined by the red lines. Most of the values are focused close to the origin which is not visible in the scatterplot. (TIFF) [file pone.0041196.s004.tiff]

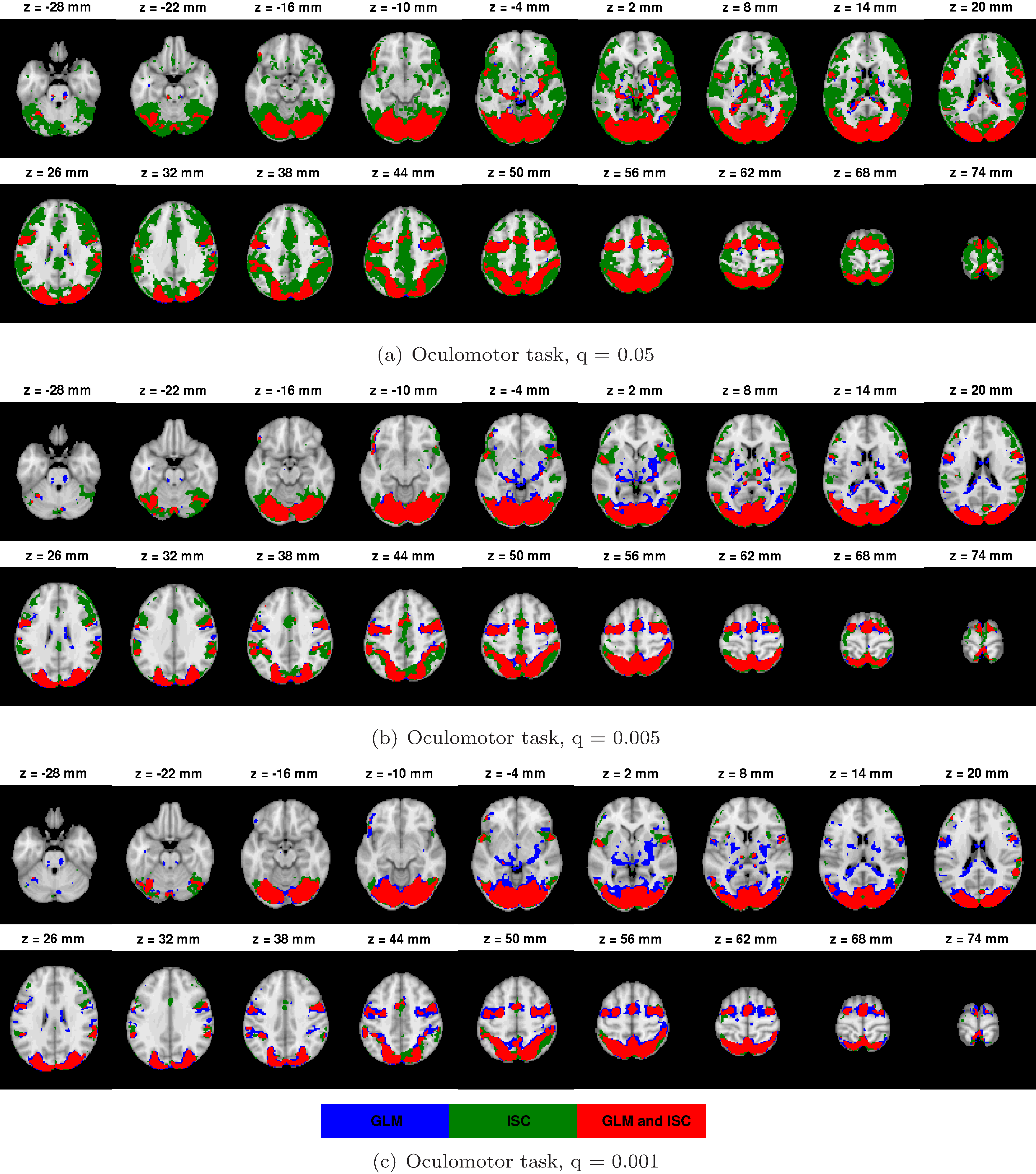

Supplement: Figure S5 — GLM and ISC analysis results for the OM task. In the image the thresholded (FDR corrected, q0.05 (a), q0.005 (b) and q0.001 (c)) ) results for OM task are presented as a binary overlay image. The color coding in the images is the same as in Figure 3 of the article. As earlier with the HA task in Figure S3, also here ISC was first very liberal q0.05 and there was mainly common (red) and ISC only (green) areas. When the threshold gets tighter q0.005 the ISC only areas becomes smaller like with AN task and with the tightest threshold q0.001 ISC becomes more conservative than GLM. Here some ISC only areas remained visible even with the tightest significance level q0.001. (TIFF) [file pone.0041196.s005.tiff]

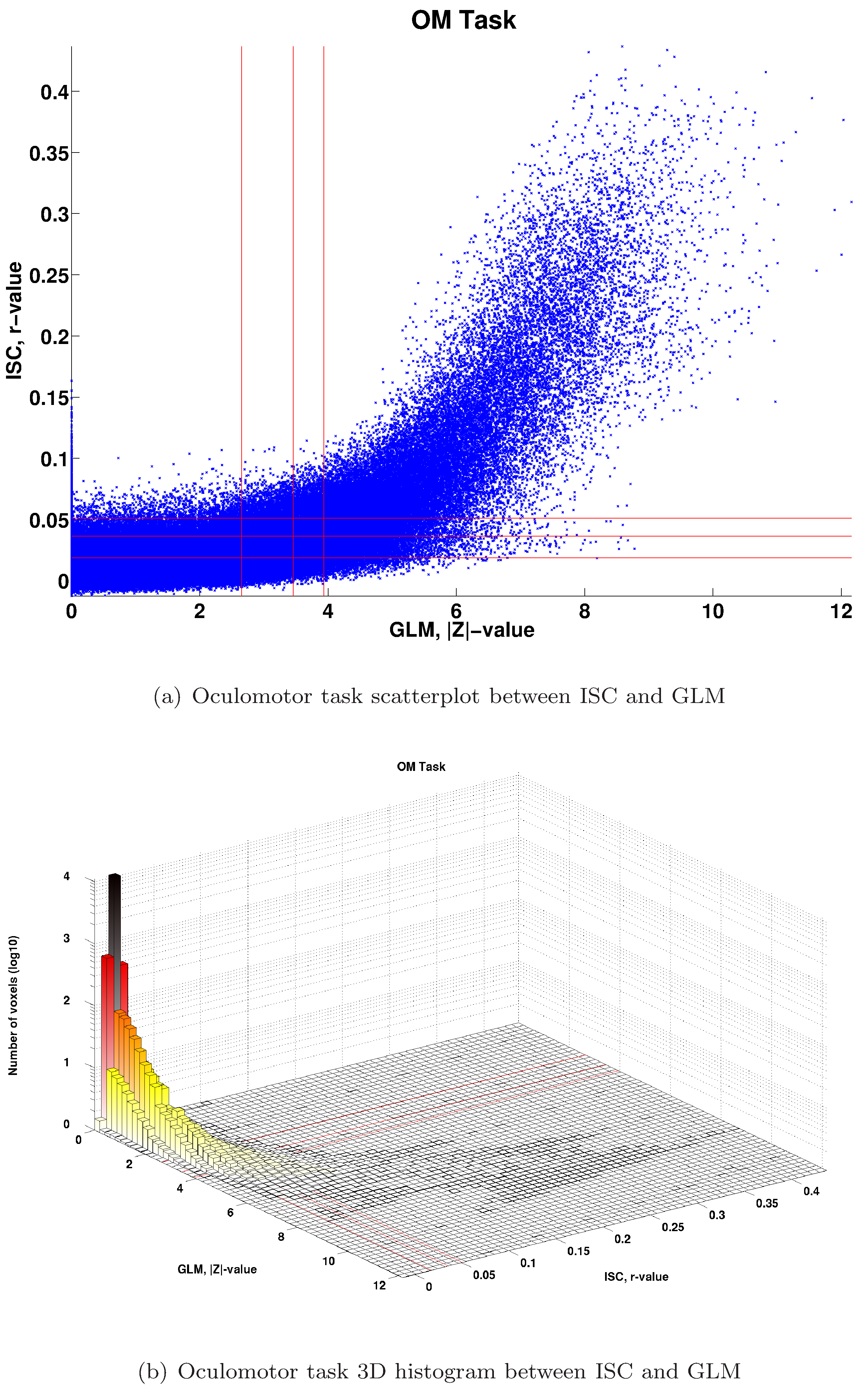

Supplement: Figure S6 — GLM and ISC analysis results for the OM task. The scatterplot (a) presents the voxel-wise statistic values of GLM (horisontal axis) and ISC (vertical axis). Red lines define the thresholds with levels q = 0.05, q = 0.005 and q = 0.001. The second image (b) displays the corresponding histogram, which shows more clearly how the mass of the values is distributed with respect to the thresholds defined by the red lines. Most of the values are focused close to the origin which is not visible in the scatterplot. (TIFF) [file pone.0041196.s006.tiff]

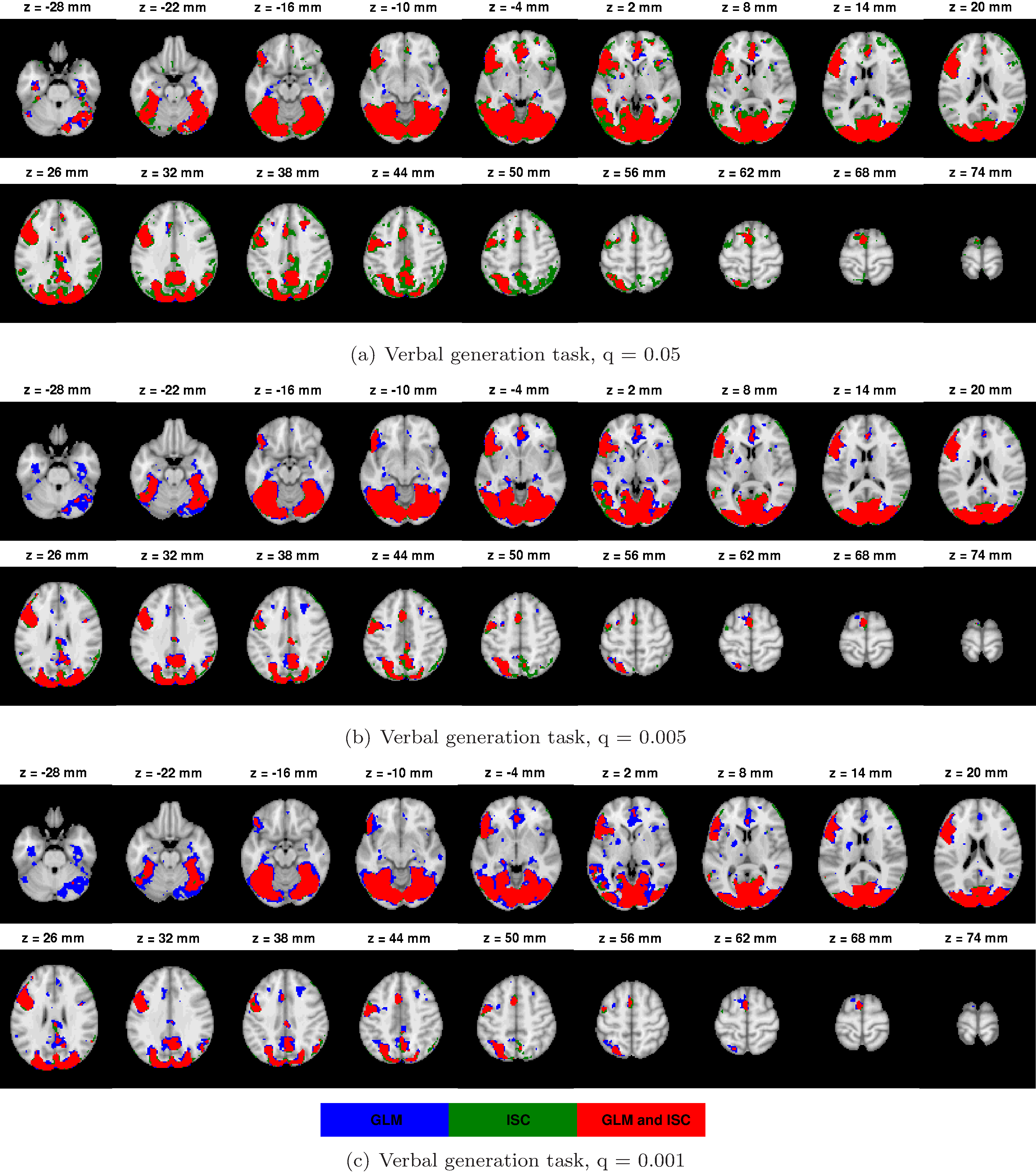

Supplement: Figure S7 — GLM and ISC analysis results for the VG task. In the image the thresholded (FDR corrected, q0.05 (a), q0.005 (b) and q0.001 (c)) results for VG task are presented as a binary overlay image. The color coding in the images is the same as in Figure 3 of the article. Here we can see the similar progress than with the task EO. There were merely a few ISC only areas (green) without GLM areas next to them and most of the common (red) areas were surrounded by GLM only areas (blue). When the threshold tightened from q0.05 to q0.001 both ISC and GLM detections contracted, but ISC contracted somewhat faster, which again suggested that ISC was more conservative than GLM. (TIFF) [file pone.0041196.s007.tiff]

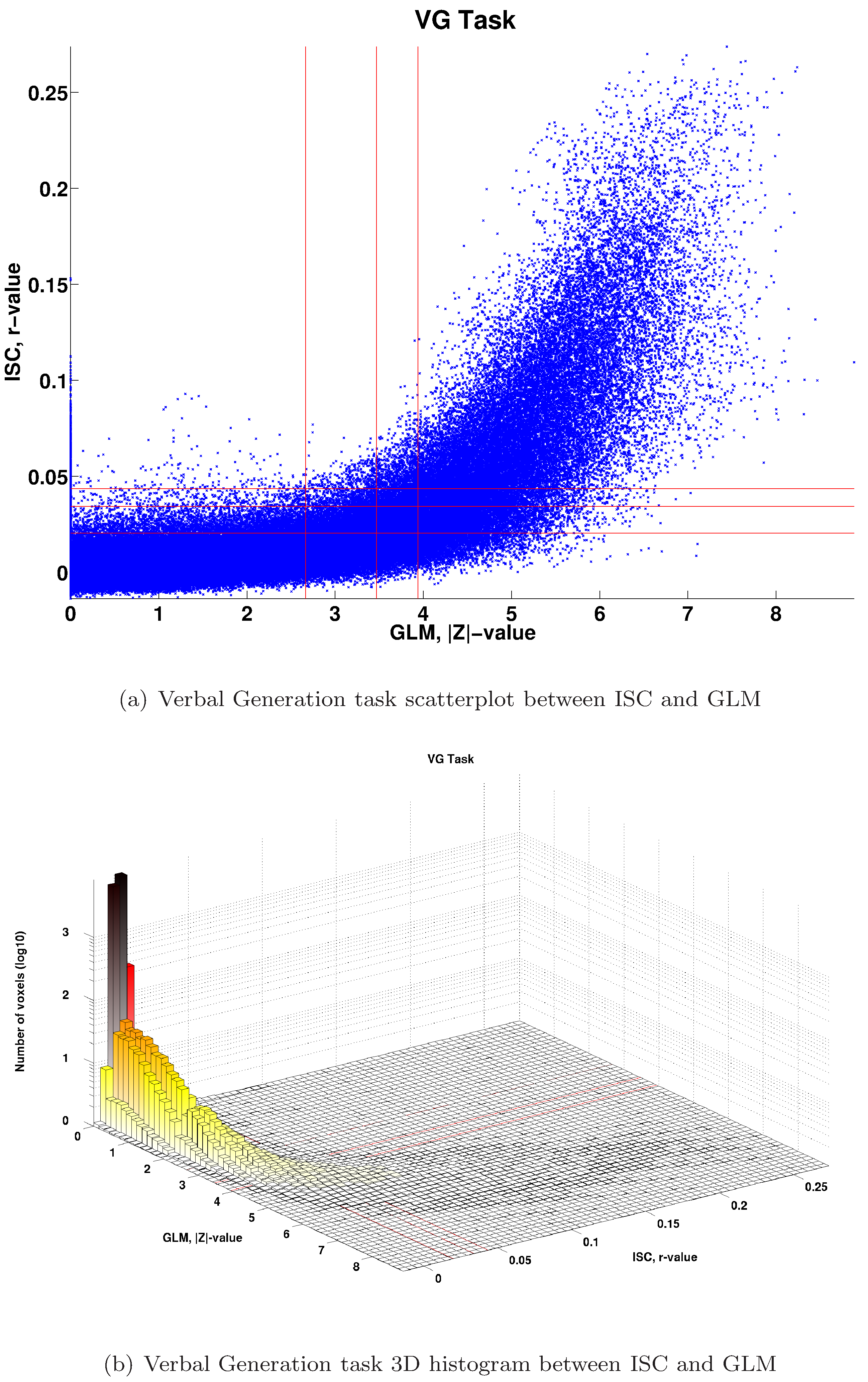

Supplement: Figure S8 — GLM and ISC analysis results for the VG task. The scatterplot (a) presents the voxel-wise statistic values of GLM (horisontal axis) and ISC (vertical axis). Red lines define the thresholds with levels q = 0.05, q = 0.005 and q = 0.001. The second image (b) displays the corresponding histogram, which shows more clearly how the mass of the values is distributed with respect to the thresholds defined by the red lines. Most of the values are focused close to the origin which is not visible in the scatterplot. (TIFF) [file pone.0041196.s008.tiff]

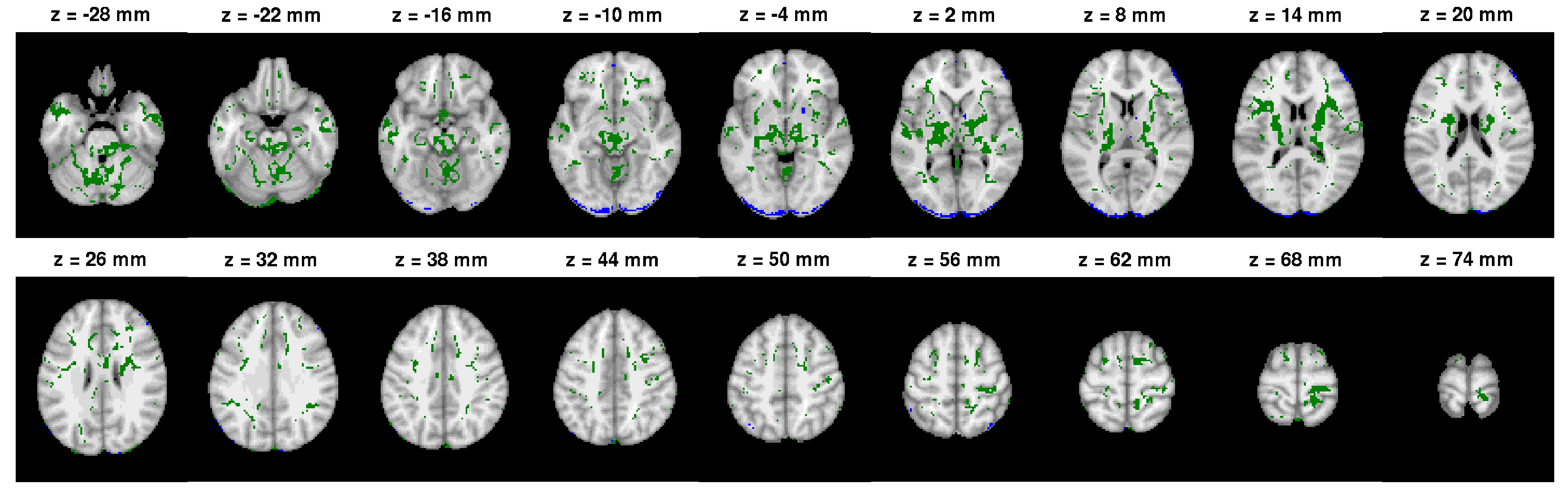

Supplement: Figure S9 — The voxels consistently detected as activated by one method and not by the other with EO task. Green color indicates voxels which were detected as activated by GLM in all thresholding levels, but not detected as activated by ISC in even the most liberal thresholding level (q = 0.05). Viceversa, blue color indicates voxels which were detected as activated by ISC in all of the thresholding levels, but not detected as activated by GLM with even the most liberal thresholding level (q = 0.05). Mostly these are isolated voxels or voxels lying near the boundary of the activation area. (TIFF) [file pone.0041196.s009.tiff]

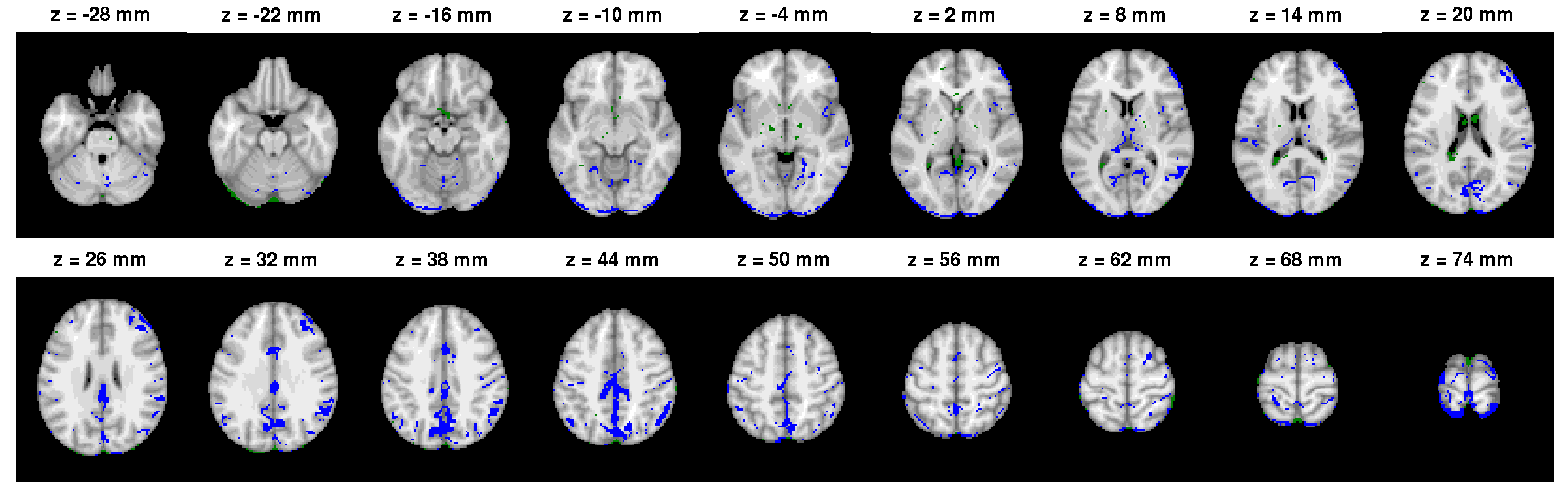

Supplement: Figure S10 — The voxels consistently detected as activated by one method and not by the other with HA task. The color coding of the image is the same as in Figure S9. Mostly these are isolated voxels or voxels lying near the boundary of the activation area. However, the ISC detected activations in Precuneous cortex that were not detected by the GLM. (TIFF) [file pone.0041196.s010.tiff]

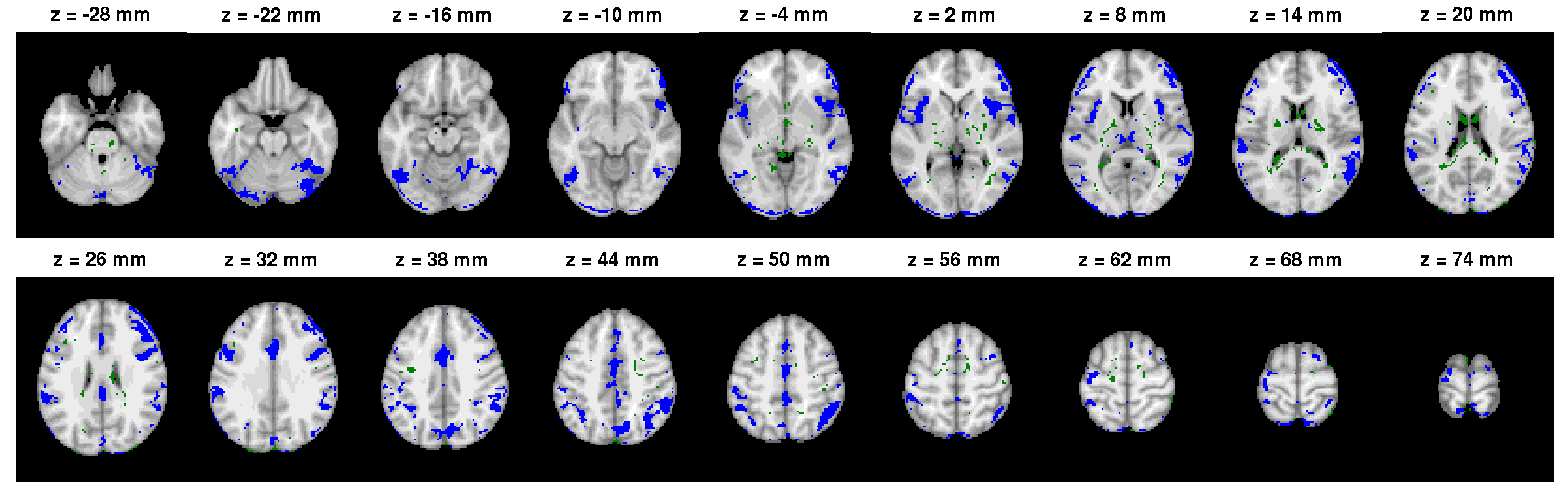

Supplement: Figure S11 — The voxels consistently detected as activated by one method and not by the other with OM task. The color coding of the image is the same as in Figure S9. Mostly these are isolated voxels or voxels lying near the boundary of the activation area. However, the ISC detected activations in middle frontal gyrus that were not detected by the GLM. (TIFF) [file pone.0041196.s011.tiff]

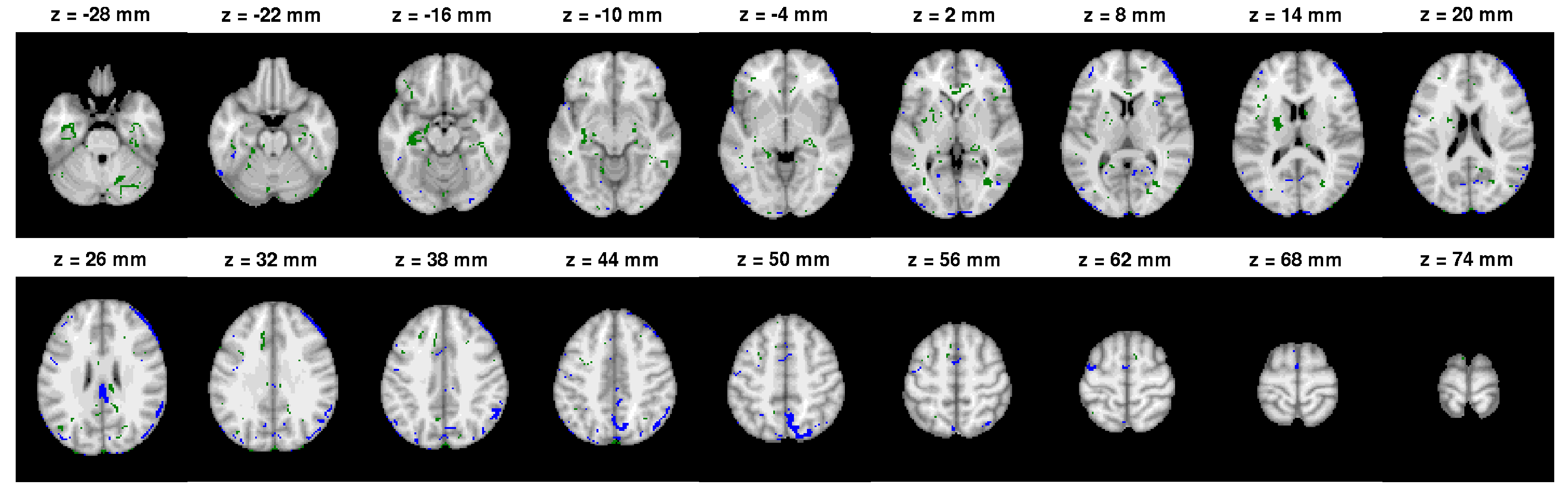

Supplement: Figure S12 — The voxels consistently detected as activated by one method and not by the other with VG task. The color coding of the image is the same as in Figure S9. Mostly these are isolated voxels or voxels lying near the boundary of the activation area. However, the ISC detected activations in middle temporal cortex and in superior cortex that were not detected by the GLM. (TIFF) [file pone.0041196.s012.tiff]
